# circPDE4B downregulation triggers GEMIN5‑dependent translational stress response and autophagy to reduce MAPT pathology

**DOI:** 10.1002/alz.71436

**Published:** 2026-05-04

**Authors:** Sambhavi Puri, Sophie J. F. van der Spek, Mintao Lin, Zihan Wang, Jacob Porter, Junming Hu, Liyuan Hu, Alejandro N. Rondón‐Ortiz, Alexander Knyshov, Jenna L. Libera, Scott E. Counts, Debomoy K. Lahiri, Julia TCW, Andrew Emili, Xiaoling Zhang, Benjamin Wolozin

**Affiliations:** ^1^ Department of Anatomy and Neurobiology Boston University Chobanian and Avedisian School of Medicine Boston Massachusetts USA; ^2^ Department of Medicine (Biomedical Genetics) Boston University Chobanian and Avedisian School of Medicine Boston Massachusetts USA; ^3^ Department of Biomedical Engineering Division of Oncological Sciences Knight Cancer Institute Oregon Health and Science University Portland Oregon USA; ^4^ Department of Pharmacology, Physiology and Biophysics Boston University Chobanian and Avedisian School of Medicine Boston Massachusetts USA; ^5^ Department of Translational Neuroscience Department of Family Medicine Michigan State University Grand Rapids Michigan USA; ^6^ Department of Psychiatry Stark Neuroscience Research Institute Indiana University School of Medicine, Indianapolis Indiana USA; ^7^ Department of Biostatistics Boston University School of Public Health Boston Massachusetts USA; ^8^ Framingham Heart Study Boston University Chobanian and Avedisian School of Medicine Boston Massachusetts USA; ^9^ Department of Neurology Boston University Chobanian and Avedisian School of Medicine Boston Massachusetts USA

**Keywords:** Alzheimer's disease, autophagy, circPDE4b, circular RNA, gem‐associated protein 5, microtubule‐associated protein tau pathology, neuronal homeostasis, translational regulation, translational stress response

## Abstract

**INTRODUCTION:**

Circular RNAs (circRNAs) are emerging as key regulators of gene expression, synaptic plasticity, and neuronal function in Alzheimer's disease (AD). Here, we characterize the biological actions of circPDE4B, a highly expressed circRNA markedly reduced in AD.

**METHODS:**

circPDE4B knockdown in neuronal progenitor cells was combined with RNA sequencing to identify regulated pathways. circPDE4B affinity purification identified major protein and micro RNA (miRNA) interactors. Assays of translation and autophagy integrated circPDE4B actions.

**RESULTS:**

We found that circPDE4B knockdown inhibited translation through a mechanism mediated by its major interacting protein, gem‐associated protein 5. circPDE4B knockdown also decreased mechanistic target of rapamycin and correspondingly enhanced autophagic flux. Consistent with these actions, circPDE4B knockdown strongly attenuated microtubule‐associated protein tau pathology in a 3D human assembloid model of tauopathy.

**DISCUSSION:**

Collectively, our findings identify circPDE4B as a regulator of neuronal homeostasis that integrates translation, autophagy, and miRNA pathways, highlighting a potentially important role in the pathophysiology of AD.

## Highlights

- circPDE4B expression is markedly downregulated across multiple brain regions in Alzheimer's disease (AD).
- circPDE4B knockdown inhibits protein synthesis through a process mediated by gem‐associated protein 5.
- circPDE4B knockdown enhances autophagic flux and ameliorates tau pathology in a 3D human brain assembloid model.
- AD‐associated micro RNAs, including miR‐124 and miR‐132, interact with circPDE4B, suggesting a regulatory network relevant to neuronal homeostasis.

## 1 BACKGROUND

Alzheimer's disease (AD) is a progressive neurodegenerative disorder and the leading cause of dementia, affecting millions worldwide. It is marked by amyloid beta (Aβ) plaques, neurofibrillary tangles, synaptic loss, and chronic neuroinflammation.^1^ Impaired autophagy further accelerates neurodegeneration by disrupting the clearance of toxic protein aggregates.^2^ These pathological processes form a self‐reinforcing cycle, highlighting the urgent need for targeted therapies and early diagnostics. While changes in the AD messenger RNA (mRNA) transcriptome are well documented, the role of non‐coding RNAs, particularly circular RNAs (circRNAs), is largely unexplored and is only beginning to emerge.

CircRNAs are a unique class of RNA molecules characterized by their covalently closed loop structure, formed through a non‐canonical back‐splicing process.^3^, ^4^ This back‐splicing is often facilitated by specific sequence motifs and RNA‐binding proteins (RBPs) that help bring splice sites together.^4^, ^5^ Their circular configuration makes circRNAs highly stable and resistant to exonuclease‐mediated degradation, with a half‐life ≈ 2.5 times longer than that of typical mRNAs.^6^, ^7^ circRNAs exhibit tissue‐ and developmental stage–specific expression patterns, highlighting their regulatory importance in various biological contexts.^8^, ^9^, ^10^ Functionally, they act as versatile regulators of gene expression by serving as microRNA (miRNA) sponges that sequester miRNAs, binding and modulating RBPs, and, in some cases, even acting as templates for translation through internal ribosome entry sites.^11^, ^12^, ^13^ The dysregulation of circRNAs has been linked to a range of diseases, including cancer, neurodegenerative disorders, and cardiovascular conditions, highlighting their potential as biomarkers and therapeutic targets.^14^, ^15^, ^16^, ^17^ Advances in bioinformatics and high‐throughput sequencing techniques have enabled the discovery and analysis of circRNAs, further establishing their significance in cellular homeostasis and disease pathogenesis.

circRNAs have emerged as key players in neurodegeneration, providing insights into diseases like AD and related dementias (ADRD), Parkinson's disease (PD), and Huntington's disease.^18^ circRNAs show neuronal and synaptic enrichment and increased expression with age, which suggests a significant role in neurodegenerative processes.^19^, ^20^, ^21^, ^22^ We and others previously characterized the circRNAs that are differentially expressed in AD brains, many of which show brain‐specific expression patterns, particularly in regions affected by ADRD.^23^, ^24^ A recent study identified 11,039 circRNAs in human brain neurons, linking 29% of circRNAs to PD and 12% to AD‐associated genes, including circAPP, consistent with our previous work.^24^, ^25^ An increasing number of studies are beginning to elucidate the roles of circRNA in neurodegeneration. For instance, circHDAC9 is downregulated in the sera of AD patients and the hippocampal tissues of AD mice. circHDAC9 normally acts as a miRNA sponge for miR‐138, which is upregulated in AD and has been shown to exacerbate Aβ toxicity.^26^ The reduced levels of circHDAC9 in AD might increase levels of free miR‐138 enhancing neurodegeneration.^27^ Similarly, *circSLC8A1* is significantly upregulated in the substantia nigra of PD patients where it sponges miR‐128, leading to increased expression of neurotoxic proteins.^28^ Many other circRNAs have been shown to affect key pathological processes associated with AD, including Aβ accumulation and microtubule‐associated protein tau (MAPT) aggregation; however, the mechanisms through which these disease‐relevant circRNAs act is largely unknown.^29^ The stability of circRNA combined with their strong effects renders them an important emerging area of neurobiology that could lead to promising novel therapeutic strategies for ADRD.

In this study, we investigate circPDE4B, which is significantly downregulated in AD across multiple brain regions, indicating its robust involvement in disease pathophysiology. circPDE4B is unusual because it is expressed at much higher levels than its linear counterpart in both human brain tissue and induced pluripotent stem cell (iPSC)‐derived neurons. Indeed, circPDE4B is the predominant circRNA isoform produced by the *PDE4B* gene, suggesting a distinct regulatory role and biological significance. Here we demonstrate a profound action of circPDE4B in neuronal pathophysiology. We show that: (1) downregulation of circPDE4B induced a translational stress response, indicating a critical role in regulating protein synthesis; (2) the protein interaction network for circPDE4B revealed gem‐associated protein 5 (GEMIN5) is the mediator of circPDE4B translational effects; (3) knockdown (KD) of circPDE4B led to reduced mechanistic target of rapamycin (mTOR) expression with a corresponding increase in autophagic flux, and importantly, was sufficient to reduce MAPT pathology in a human 3D iPSC‐derived assembloid model of tauopathy; and (4) AD‐associated miRNAs interact with circPDE4B, including miR‐124 and 132, that exhibit broad regulatory influence across multiple molecular pathways implicated in AD.

## 2 METHODS

### 2.1 Key resources and reagents

Details of all reagents, kits, antibodies, and software used in this study are provided in Table S1 in supporting information.

### 2.2 circRNA analysis from AD brain RNA sequencing dataset

We analyzed circRNAs from publicly available human *post mortem* brain RNA sequencing (RNA‐seq) resources from the Accelerating Medicines Partnership for Alzheimer's Disease: the Mount Sinai Brain Bank (MSBB; Synapse syn315774), spanning the frontal pole (Brodmann area [BM]10), the superior temporal gyrus (BM22), the parahippocampal gyrus (BM36), and the inferior frontal gyrus (BM44). Samples flagged by provider metadata as failing quality or processing checks were excluded, as were individuals with missing key covariates; remaining libraries were screened using alignment and library‐level metrics. circRNAs were called and quantified as described in previously published methods.^24^, ^30^ Expression matrices used log2(FPB + 0.5) and were filtered to retain circRNAs detected in > 50% of samples within each region and cohort. Differential expression between AD and control was evaluated separately for each brain region and cohort using linear models implemented in limma with empirical Bayes moderation, adjusting for sex, age or age at death, RNA quality metrics (e.g., RNA integrity number/RNA quality number), *post mortem* interval (PMI) where available, and library/batch; *p* values were corrected by the Benjamini–Hochberg method to yield region‐ and cohort‐specific lists.^31^ After differential expression (DE) analysis and false discovery rate (FDR) correction, we compared significant circRNA sets across the four MSBB cortical regions (BM10, BM22, BM36, BM44) using set intersections/unions and summarized overlaps with Venn diagrams.

**RESEARCH IN CONTEXT**

1. **Systematic review**: Several studies, including our own, have reported widespread dysregulation of circular RNAs (circRNAs) in Alzheimer's disease (AD) and related dementias (ADRD), supporting a role for these molecules in disease pathogenesis. circPDE4B is one such circRNA that is consistently downregulated in AD post mortem brain tissue. circPDE4B is unusual because its expression in the brain is ≈ 3‐fold more than that of its cognate messenger RNA. circRNAs are increasingly recognized as regulators of neuronal function and synaptic plasticity through their interactions with micro RNAs (miRNAs) and RNA‐binding proteins, positioning them as key modulators of post‐transcriptional gene control. circRNAs are enriched in the brain and accumulate with aging, linking their dysregulation to neurodegenerative processes and reinforcing their relevance to AD mechanisms.
2. **Interpretation**: circPDE4B is downregulated across multiple brain regions in AD, suggesting a role in the pathophysiology of AD. Our studies identify key molecular pathways that are regulated by circPDE4B and are known to contribute to the pathophysiology of AD. When circPDE4B is reduced, it broadly decreases RNA translation through a process mediated by gem‐associated protein 5, a major interactor; decreased RNA translation commonly reflects an adaptation to chronic stress. In parallel, circPDE4B downregulation enhances autophagy, which in turn promotes clearance of pathological protein aggregates, aligning circPDE4B‐driven pathways with key pathways that could be therapeutic in neurodegeneration.
3. **Future directions**: Our findings provide a strong mechanistic framework for how circPDE4B contributes to the pathophysiology of AD. Extending these studies to 3D assembloid models will define how circPDE4B shapes neuron–glia crosstalk and circuit‐level vulnerability in AD. In parallel, dissecting its interactions with specific miRNAs and their downstream targets should clarify the broader post‐transcriptional networks engaged by circPDE4B, ultimately deepening understanding of AD mechanisms and potential therapeutic entry points. Finally, future studies will examine the effects of circPDE4B knockdown in vivo, which allows determination of the impact of circPDE4B on neuronal resilience and proteostasis in a more complex environment that incorporates aging. Together these studies could test the benefits of circPDE4B knockdown as a potential therapeutic approach for ADRD.

### 2.3 Tissue samples

*Post mortem* brain tissues were obtained from the University of Kentucky Alzheimer's Disease Research Center (UK‐ADRC) at the Sanders Brown Center on Aging. Dorsolateral prefrontal cortex (BM9) samples from control cases (*n* = 15) were used in this study. The demographics, clinical characteristics, and PMIs of the cases are presented in Table S2 in supporting information. Human subject consent was not required for this study.

### 2.4 Cell culture and lentivirus production

Purified control male individual line of neuronal progenitor cells (NPCs) were generously provided by Dr. Julia TCW.^32^ These NPCs were cultured in STEMdiff Neural Progenitor Medium (StemCell Technologies, Cat# 05833) on plates pre‐coated with Matrigel and maintained in a humidified incubator at 37°C under an atmosphere of 5% CO_2_. For lentivirus production, HEK‐293T cells were seeded into 6‐well dishes and allowed to reach ≈ 60% to 80% confluency. The HEK‐293T cells were then transfected with a mixture of plasmids consisting of the transfer plasmid, psPAX2 (Addgene, Cat#12260), and VSV‐G (Addgene, Cat#8454) at a ratio of 3:2:1, respectively, using Fugene‐HD transfection reagent (Promega, Cat# E2311). Three days after transfection, the supernatant containing lentiviral particles was harvested. The viral supernatant was concentrated using the Lenti‐X Concentrator (Takara Bio USA, Cat# 631232) according to the manufacturer's instructions. Finally, the lentiviral titer was determined using the Lenti‐X p24 Rapid Titer Kit (Takara Bio USA, Cat# 632200).

### 2.5 Nuclear and cytoplasmic fractionation

NPCs were pelleted and washed thrice in ice‐cold 1X phosphate buffered saline (PBS). Cells were lysed on ice in cytoplasmic lysis buffer (10 mM Tris pH 7.9, 1.5 mM MgCl2, 10 mM KCl, 0.2% NP40, 1X protease inhibitor cocktail and RNase Inhibitor) for 10 minutes. The nucleus was sedimented by centrifugation at 2500 × g for 5 minutes, and the supernatant containing cytoplasm was removed. The nuclear pellet was rewashed in cytoplasmic lysis buffer to remove any contaminating cytoplasm. The RNA from cytoplasmic fraction and nuclear fraction was isolated using Trizol extraction method discussed below. U6 and glyceraldehyde 3‐phosphate dehydrogenase were used as nuclear and cytoplasm controls, respectively.

### 2.6 BaseScope

NPCs were seeded into 8‐chamber well plates and cultured until they reached 60% to 70% confluence. The cells were then fixed with freshly prepared 4% paraformaldehyde for 30 minutes at room temperature (RT), followed by a series of dehydration and rehydration steps with 100%, 70%, and 50% ethanol in preparation for BaseScope (ACD Bio, Cat# 323800) analysis. The BaseScope procedure was conducted according to the manufacturer's instructions. Briefly, after fixation, cells were treated with hydrogen peroxide for 10 minutes at RT, followed by Protease III for another 10 minutes at RT to facilitate probe access, with washes between each step. Subsequently, cells were incubated with a circPDE4B‐specific probe targeting the backsplicing junction (target sequence in Table S3 in supporting information) for 2 hours at 40°C in a hybridization oven using a humidity control tray. Signal amplification steps were then performed using eight sequential amplifiers (v2 AMP 1‐8) supplied in the kit, with each amplification reagent applied for the specified incubation time at 40°C or RT, followed by wash steps between incubations. Finally, cells were stained with BaseScope Fast RED for 10 minutes at RT protected from light, to visualize the target RNA. The cells were then washed with 1X PBS and counterstained with 4′,6‐diamidino‐2‐phenylindole. Immunofluorescence images were acquired using a Zeiss LSM700 laser‐scanning confocal microscope with a 63× oil immersion objective. The pinhole size was maintained at 1 Airy Unit, and images were captured using Zen Black software from Zeiss.

### 2.7 Plasmids, transduction, and treatments

To downregulate circPDE4B in NPCs, a single guide RNA (sgRNA) sequence targeting the backsplicing junction of circPDE4B (Table S3) was cloned into the pLentiRNACRISPR_006 vector (Addgene, Cat#138148), which features the hU6‐DR_BsmBI‐EFS‐RfxCas13d‐NES‐2A‐Puro‐WPRE construct. NPCs were seeded and allowed to reach 60% to 70% confluence. The cells were then transduced with either control or circPDE4B‐targeting sgRNA lentiviral particles for 48 hours. After transduction, cells were selected with 0.5 µg/mL puromycin for 4 days. After selection, the cells were harvested for subsequent downstream experiments.

To evaluate autophagic flux, NPCs after circPDE4B KD were subjected to the following treatment conditions: (1) incubation in complete NPC media, (2) incubation in complete NPC media supplemented with 100 nM bafilomycin A1 (BAFA1; Tocris, Cat#1334) for 4 hours, (3) incubation in Hank's Balanced Salt Solution (HBSS) for 4 hours, and (4) incubation in HBSS with the addition of 100 nM BAFA1 for 4 hours. After the respective treatments, cells were harvested and lysed. The resulting lysates were subjected to immunoblotting to assess LC3B regulation as an indicator of autophagy.

### 2.8 RNA isolation and real time qualitative polymerase chain reaction

NPCs were lysed in 1 mL of TRIzol using a motor‐driven pestle. The resulting lysate was incubated on ice for 5 minutes, after which 200 µL of chloroform was added. The samples were vortexed at medium speed for 15 seconds and then kept on ice for an additional 3 minutes. Phase separation was achieved by centrifugation at 12,000 × g for 15 minutes at 4°C. The aqueous layer containing RNA was carefully removed and purified using the Qiagen RNeasy Plus Mini Kit (Qiagen, Cat# 74134) according to the manufacturer's instructions. RNA was eluted in 30 µL of RNase‐free water and stored at −80°C until further use.

For complementary DNA (cDNA) synthesis, RNA was reverse transcribed using the High‐Capacity cDNA Reverse Transcription Kit (Applied Biosystems, Cat# 4368814) following the provided protocol. For amplification of circPDE4B, divergent primers across the back‐splice junction were used. All primer sequences are listed in Table S2. Each sample was analyzed in triplicate or quadruplicate technical replicates for quantitative polymerase chain reaction (PCR). Reactions were performed on a QuantStudio 12K Flex quantitative PCR (qPCR) system. Cycle threshold (Ct) values were compared using the ΔΔCt method to determine relative gene expression.

### 2.9 Total RNA sequencing

RNA was isolated from cells using the protocol listed above. The sequencing libraries were prepared using SMART‐Seq Total RNA High Input kit following manufacturer's instructions (Takara Bio USA, Inc., Cat# 635046). Briefly, 100 ng of total RNA was subjected to fragmentation and ribosomal RNA (rRNA) depletion followed by first‐strand cDNA synthesis. Subsequently, the libraries were barcoded with dual indexes and purified using AMPure beads. The cDNA libraries underwent validation through the Agilent Bioanalyzer and were then subjected to sequencing on an Illumina NextSeq 2000, yielding a read depth of > 30 million reads per sample.

Raw sequencing reads underwent standard quality control using FastQC and MultiQC. Clean reads were aligned to the Homo sapiens GRCh38 reference genome with STAR (v2.7.10a) in two‐pass mode. Gene and transcript abundance were quantified using RSEM (v1.3.3), yielding expected counts and transcripts per million (TPM) values for each sample. Samples with > 85% uniquely mapped reads were retained for downstream analysis. Gene‐level count data were imported into R (v4.3.0) using the tximport package and normalized with DESeq2 (v1.40.2). Principal component analysis (PCA) was performed on variance‐stabilized transformed counts to assess inter‐sample variability (Figure S1 in supporting information). Differential expression analysis was conducted using the Wald test in DESeq2 with Benjamini–Hochberg correction (adjusted *p* < 0.05).

### 2.10 Immunoblotting

For all immunoblotting experiments, cells were lysed in RIPA buffer (50 mM Tris‐HCl, pH 8.0, 150 mM sodium chloride, 1.0% Igepal CA‐630 [NP‐40], 0.5% sodium deoxycholate, and 0.1% sodium dodecyl sulfate) supplemented with protease inhibitor (Thermo Fisher, Cat# A32955) and phosphatase inhibitor cocktail (Sigma, #PHOSS‐RO), unless otherwise specified. After lysis, cells were incubated on ice for 30 minutes and subsequently centrifuged at 15,000 g for 30 minutes at 4°C. The supernatant was carefully collected and protein concentration was determined using the bicinchoninic acid assay. For each sample, 15 to 50 µg of total protein (depending on the experiment) was resolved by sodium dodecyl sulfate polyacrylamide gel electrophoresis (SDS‐PAGE) and transferred to a membrane for western blot analysis. All antibodies for western blot were resuspended in blocking buffer (5% milk in 0.1% PBST).

### 2.11 Bio‐orthogonal non‐canonical amino acid tagging assay

Cells were plated in 12‐well dishes and cultured until they reached 100% confluence. Upon reaching confluence, the cells were gently washed with PBS and then incubated in methionine‐free medium (Thermo Fisher Cat# 21013024 supplemented with L‐glutamine and sodium pyruvate) at 37°C for 60 minutes to deplete endogenous methionine reserves. After methionine depletion, 100 µM L‐homopropargylglycine (HPG) was added to the methionine‐free culture medium, and the cells were incubated for an additional hour at 37°C to allow metabolic incorporation of HPG into newly synthesized proteins. After the labeling period, cells were harvested and lysed in RIPA buffer. Protein lysates were labeled using the Click‐&‐Go Protein Reaction Buffer Kit (Vector Labs, Cat# CCT‐1262) following the manufacturer's protocol. Briefly, 50 µL of cleared protein lysate (1 µg/µL total protein in RIPA buffer, containing 1% SDS) was combined with the 100µl of Component A consisting of 40uM azide–biotin detection reagent. This was followed by addition of Copper(II) Sulfate (Component B) and the reducing agent (Component C) to initiate the copper‑catalyzed azide–alkyne cycloaddition between HPG‐labeled proteins and the azide–biotin probe. The mixture was vortexed briefly after each addition, protected from light, and incubated for 30 minutes at RT to allow complete conjugation. After click labeling, proteins were repurified by methanol–chloroform precipitation. Six hundred µL of methanol were added to the 200 µL reaction, followed by 150 µL of chloroform and 400 µL volumes of water, with vigorous vortexing after each addition to ensure phase separation. Samples were centrifuged at 18,000 × g for 5 minutes at RT to form a visible interphase protein disk, after which the upper aqueous phase was carefully discarded without disturbing the protein layer. An additional 450 µL volumes of methanol were then added to the remaining lower organic phase and interphase, samples were vortexed vigorously, and centrifuged again at 18,000 × g for 5 minutes to pellet the proteins. The supernatant was discarded, and pellets were air dried at RT and resuspended in in 2X SDS Laemmli buffer (BioRad, Cat#1610747) by vigorous pipetting and heating at 95°C for 10 minutes. Samples were resolved by SDS‐PAGE and proteins were transferred to a membrane and probed with streptavidin–horseradish peroxidase conjugate, allowing visualization of biotinylated, newly synthesized proteins by western blot.

### 2.12 Affinity purification of circPDE4B

NPCs were grown in 6‐well plates until 100% confluency. For affinity purification (AP) of circPDE4B:protein complexes, cells were crosslinked by UV (4000 × 100 µJ/cm^2^, three times; Stratalinker 2400 UV Crosslinker, Stratalinker) before lysis. No crosslinking was performed for AP of circPDE4B:mRNA complex. APs were performed as previously described by Li et al. with several adaptations.^33^ In short, per AP experiment, two wells were washed with PBS, lysed in 1 mL total lysis buffer (150 mM NaCl, 1 mM EDTA, 0.5% NP40, 20 mM Tris, pH 7.5, supplemented with 250 U/AP RNAse inhibitor [NEB, Cat#M0314L] and 0.5× protease inhibitor [Halt, Thermo Fisher, Cat# 78429]), and transferred to a collection tube. Lysate was then sonicated three times at constant speed for 10 seconds followed by 1‐minute cooling on ice (60 Sonic dismembrator, Thermo Fisher, setting 6), and additional RNAse and protease inhibitor was spiked in (to a 500U/AP and 1x end ‐concentration, respectively). Per AP experiment, 400 pmol biotinylated PDB4E‐probe (5′AACACTTTTTAATACCACTCCTGGCTTACA/3BioTEG/‐3, custom‐made, IDT) or Scrambled probe (5′GACAATCTTCCAAATATCGCTTTACTCTCA/3BioTEG/‐3, custom‐made, IDT) was preheated at 95°C for 10 minutes, followed by cooling on ice for 5 minutes. Cell lysates were centrifuged (12000 × g, 10 minutes, 4°C), supernatants were mixed and split across sample replicates, and incubated with probe for 2 hours at RT while rotating.

Per AP, 50 µL of Pierce Streptavidin Magnetic Beads (Thermo Fisher, Cat# 88816) were prewashed two times with wash buffer (150 mM NaCl, 1 mM EDTA, 0.05% NP40 and 20 mM Tris, pH 7.5), once with buffer A (100 mM NaOH, 50 mM NaCl) and twice with buffer B (100 mM NaCl). After sample incubation with beads for 1 hour at 4°C, rotating, beads were magnetized and washed with 0.5 mL wash buffer four times. For mass spectrometry, samples were instead washed two times with wash buffer followed by two washes with 50 mM ammonium bicarbonate. During the last wash, beads were transferred to clean tubes, supernatant was removed, and beads were processed according to their follow‐up application.

### 2.13 Quantitative liquid chromatography tandem mass spectrometry and data analysis

AP beads were incubated with 1 µg trypsin (Thermo Fisher Scientific, Cat# 90058) in 60 µL of 50 mM ammonium bicarbonate, overnight at 37°C, rotating. Samples were then desalted using C18 Stage tips, dried by SpeedVac, and stored at −80°C until further use.

Peptides were dissolved in 15 µL of 0.1% formic acid and loaded onto a reverse‐phase trap column (75 µm × 2 cm, Acclaim PepMap100‐C18, Thermo Scientific). Peptides were separated using an EASY‐Spray PepMap Neo UHPLC column (75 µm i.d. × 50 cm, 2 µm, 100 Å; ES75500PN, Thermo Scientific) on a Neo Vanquish UHPLC system (Thermo Scientific). Separation was done using a linear acetonitrile gradient (0%–3.2% over 6.5 minutes, then to 31.2% over 103.5 minutes) at a 0.3 µL/minute flow rate. After electrospray ionization, peptides were analyzed on an Orbitrap Exploris 480 mass spectrometer in data‐dependent mode, using one mass spectrometer (MS; m/z 350‐1450) followed by tandem mass spectrometry (MS/MS) with a normalized automated gain control target of 300% in MS1 and 50% in MS2, an intensity threshold of 5.0e4, and a 45 second exclusion window. Each MS/MS spectra file was computationally split into three files based on the FAIMS compensation voltage −50, −57, and −64. The MS/MS data were searched against the human proteome (UP000000589_10090, 2023_01_25) with MaxQuant software (v2.1.4.0), with parameters set to unique and razor peptides for protein quantification, variable modifications of methionine oxidation and N‐terminal acetylation, carbamidomethylation as a fixed modification, and trypsin digestion.

Data quality control and differential testing was performed using the Mass Spectrometry Downstream Analysis Pipeline (MS‐DAP, v1.0.3.1).^34^ Peptides present in ≥ 60% of samples per condition were used for differential testing by DEqMS after rollup to proteins. The Variance Stabilizing Normalization and mode between protein algorithms were used for normalization. Proteins detected in ≥ 60% of circPDE4B APs and ≤40% of Scrambled‐APs showing an abundance difference of ≥ 30% were also considered true circPDE4B interactors and included in follow‐up analyses. A PCA plot of proteomics profiles from circPDE4B affinity pulldown is shown in Figure S2 in supporting information. All raw data were deposited to the ProteomeXchange Consortium via the PRIDE partner repository^35^, ^36^ with the dataset identifier PXD070615.

### 2.14 Small RNA sequencing and data analysis

AP‐captured beads were processed for RNA extraction by resuspending in TRIzol reagent and vortexing for 30 seconds, followed by the addition of 200 µL chloroform. Samples were vortexed at medium speed for 15 seconds and incubated on ice for 3 minutes. Phase separation was carried out by centrifugation at 12,000 × g for 15 minutes at 4°C. The aqueous phase was carefully collected, and total RNA (including small RNAs/miRNAs) was purified using the NucleoSpin miRNA kit (Takara Bio USA, Cat# 740971.250) according to the manufacturer's protocol. RNA was eluted in 30 µL RNase‐free water, and quality assessment was performed using an Agilent TapeStation to confirm the presence of small RNA species.

Small RNA libraries were generated using the SMARTer smRNA‐Seq Kit for Illumina (Takara Bio USA, Cat# 635030) following the manufacturer's instructions. Briefly, 30 ng of total RNA was polyadenylated, followed by first‐strand cDNA synthesis. Amplified cDNA was subsequently ligated to full‐length Illumina sequencing adapters by PCR. PCR products were purified with the NucleoSpin Gel and PCR Clean‐Up Kit (Macherey‐Nagel Cat# 740609.50), and libraries were size‐selected with AMPure beads to enrich for fragments < 150 bp. Library size distribution and quality were validated using a TapeStation before sequencing. Libraries were sequenced on an Illumina NextSeq 2000 platform, yielding > 10 million reads per sample.

Raw small RNA reads were processed using Cutadapt (v4.3) to remove 3′ adapters and low‐quality bases, retaining reads ≥ 15 nt. Read quality was assessed with FastQC (v0.11.9), and summarized reports were generated with MultiQC (v1.14). Only libraries with > 90% of bases exceeding Phred 30 were included for downstream analysis.

High‐quality reads were quantified using miRge3.0 (v0.1.4) against the Homo sapiens miRBase v22 reference. The pipeline produced read count matrices and normalized expression values (reads per million [RPM]). Count data were imported into R (v4.3.0) and analyzed using the edgeR and limma‐voom packages. Low‐abundance miRNAs (< 5 CPM in fewer than half of the samples) were excluded. Normalization factors were estimated using the trimmed mean of M‐values (TMM) method. Variance‐stabilized expression values were computed via voom transformation, followed by linear modeling and empirical Bayes moderation to identify differentially expressed miRNAs between experimental groups. Statistical significance was defined as |log_2_ fold change| > 1 and Benjamini–Hochberg adjusted *p* < 0.05. PCA was conducted on voom‐transformed data to visualize inter‐sample relationships (Figure S3 in supporting information).

Significant miRNAs were subjected to target and network analyses. Predicted mRNA and circRNA targets were retrieved using the multiMiR database (integrating TargetScan, miRTarBase, and miRDB) and filtered for high‐confidence interactions. miRNA–mRNA and miRNA–circRNA relationships were visualized using igraph and ggraph, in which node size reflected degree centrality and edge thickness denoted interaction confidence. Hub nodes were identified by topological metrics (degree > 5 or betweenness > 0.05). Functional enrichment of target genes was assessed using clusterProfiler with Gene Ontology and Kyoto Encyclopedia of Genes and Genomes (KEGG) pathways, highlighting modules associated with neuronal signaling and synaptic regulation.

### 2.15 iPSC‐assembloid generation

For MAPT pathology experiments, we followed our previously published protocols, with the modification that iPSC‐derived MAPT S305I cells were used in place of wild‐type NPCs. The MAPT S305I line was selected as it produces robust MAPT pathology and expresses both three‐repeat and four‐repeat MAPT.^37^ Differentiation into NPCs was performed using the STEMdiff protocol (Stemcell Technologies). NPCs were transduced with either control or circPDE4B‐targeting sgRNA lentivirus for 48 hours, followed by puromycin selection for 4 days. Cells were then exposed to 0.04 mg/mL oligomeric MAPT/Tau (oTau) for 24 hours before incorporation into asteroid cultures.

Neuronal induction was achieved by transducing NPCs with a NEUROG2‐expressing lentivirus (Genocopia, Cat# LPP‐T7381‐Lv105‐A00‐S) for 24 hours, generating hiNCs, which were combined with iPSC‐derived astrocytes (hiACs) as described previously.^38^ Briefly, hiNCs and hiACs were dissociated with Accutase, washed once in DMEM/F12 (Stemcell Technologies, Cat# 36254), and mixed at a 1:1 ratio in Asteroid Medium composed of DMEM/F12 supplemented with 1% GlutaMAX (Thermo Scientific, Cat# 35050061), 1% sodium pyruvate (Thermo Scientific, Cat #11360070), 1% N‐2 supplement (Thermo Scientific, Cat# 17502‐048), 1% B‐27 supplement (Thermo Scientific, Cat# 17504044), 10 µM Y‐27632 (EMD Millipore, #SCM075), 1% penicillin–streptomycin (Thermo Scientific, Cat #15140148), and 1 mg/mL heparin (Sigma‐Aldrich, Cat# H3149‐250KU). Cell suspensions were plated into AggreWell 800 microwells pretreated with Anti‐Adherence Rinsing Solution (Stemcell Technologies, Cat# 07010), centrifuged at 100 × g for 3 minutes to promote aggregation, and incubated for 24 hours. Media was replaced by half‐volume changes at 24 hours and subsequently every other day for 3 weeks. For sample preparation for immunofluorescence labeling and immunoblotting, we followed our previously published protocol.^38^ Immunofluorescence images were acquired on a Zeiss LSM 700 laser‐scanning confocal microscope equipped with a 40× oil immersion objective.

### 2.16 Experimental replicates and statistical design

For total RNA sequencing experiments, we used three independent biological replicates per condition (control and circPDE4B KD). Each replicate corresponded to an independently cultured and transduced NPC line, and all replicates for a given experiment were processed in parallel (transduction, culture, RNA extraction, library preparation, and sequencing) to minimize batch effects. The sample size “n” for each experiment, defined as independent biological replicates, is indicated in the figure legends. For the circPDE4B AP followed by proteomics, five independent biological replicates were performed for both the scrambled control AP and the circPDE4B AP. Likewise, for small RNA sequencing, five independent biological replicates were generated for each condition (control and circPDE4B KD). All samples were processed together in a single batch for either MS or RNA extraction, library preparation, and sequencing. In all experiments, circRNA KD and matched control samples (non‑targeting control) were handled as pairs: cells were transduced and cultured in parallel, harvested on the same day, and processed side by side through all downstream steps to control for potential batch effects.

All statistical analyses were performed in GraphPad Prism (v10.5.0). Normality of data distributions was verified using the Shapiro–Wilk test. For comparisons between two groups, unpaired two‐tailed Student *t* test was applied. For comparisons among more than two groups, one‐way analysis of variance (ANOVA) followed by Šidák post hoc multiple comparison was used when ANOVA reached statistical significance (*α* = 0.05). For experiments involving two independent variables, two‐way ANOVA followed by a Šidák post hoc multiple comparison test was performed. When data did not meet normality or homogeneity assumptions, equivalent non‐parametric tests were used. Data are presented as mean ± standard error of the mean. No data points were excluded as outliers. *p* values ranges are provided in the figure legends.

## 3 RESULTS

### 3.1 CircPDE4B is consistently downregulated in AD across multiple brain regions

**FIGURE 1:**
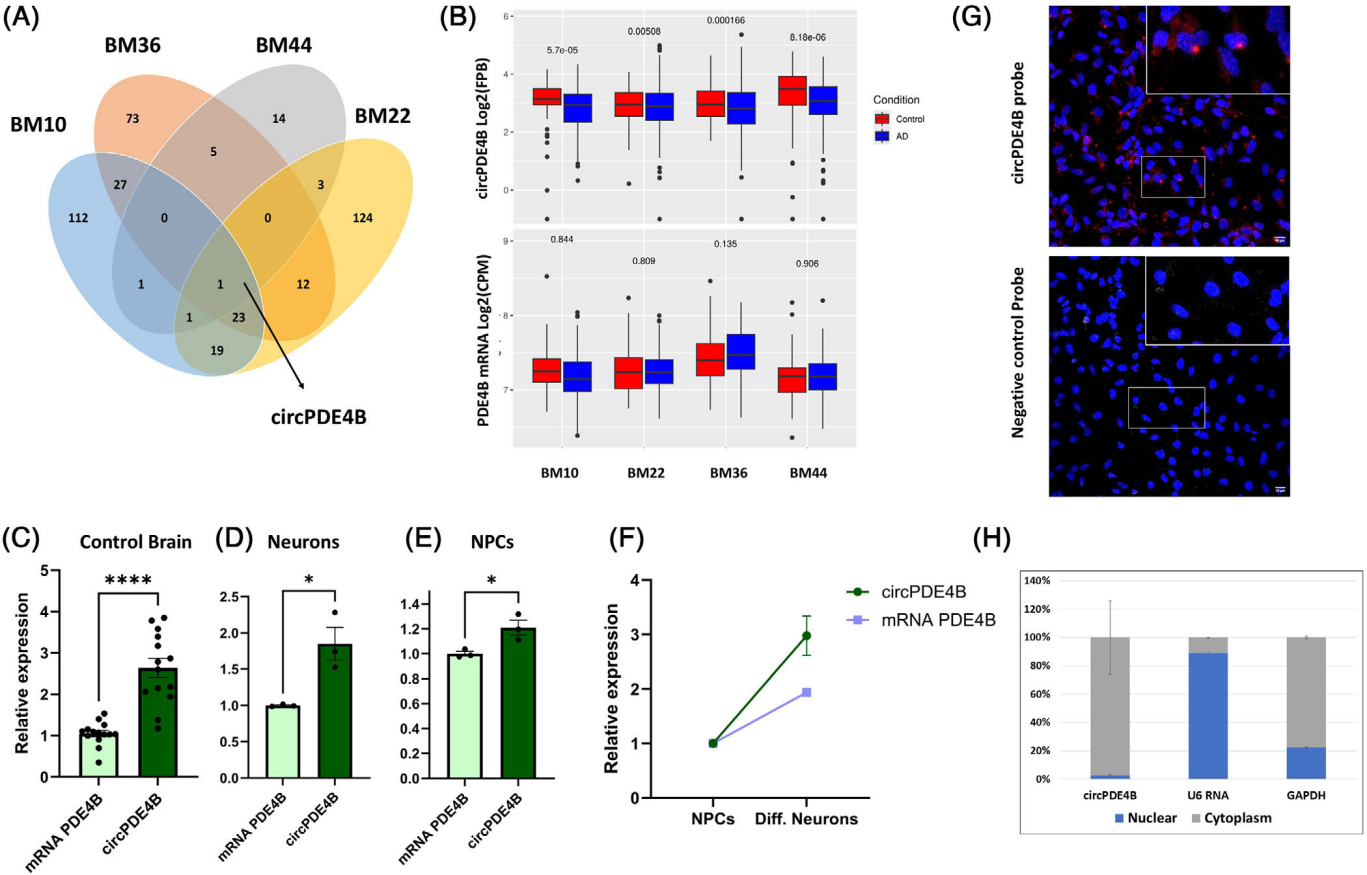
circPDE4B expression in AD. A, Venn diagram showing overlap of differentially expressed circRNAs across four brain regions in the MSBB dataset. B, Expression of circPDE4B compared to linear PDE4B mRNA in MSBB brain regions. C, qPCR analysis of circPDE4B versus linear PDE4B in *post mortem* control brain samples (Kentucky cohort, *n* = 15 biological replicate). D, qPCR expression of circPDE4B versus linear PDE4B in differentiated neurons (*n* = 3 biological replicate). E, qPCR expression of circPDE4B versus linear PDE4B in neural progenitor cells (NPCs, *n* = 3). F, Expression changes of circPDE4B and linear PDE4B during neuronal differentiation (*n* = 3, biological replicate). G, Basescope in situ hybridization showing circPDE4B expression in NPCs (red, circPDE4B probe; blue, 4′,6‐diamidino‐2‐phenylindole). H, qPCR of subcellular fractions demonstrating circPDE4B enrichment in the cytoplasm (*n* = 3, biological replicate). Data are presented as mean ± standard error. *p* values are indicated; *p < 0.05, ****p < 0.0001 by unpaired Student *t* test. AD, Alzheimer's disease; BM, Brodmann area; circRNA, circular RNA; GAPDH, glyceraldehyde 3‐phosphate dehydrogenase; mRNA, messenger RNA; MSBB, Mount Sinai Brain Bank; NPC, neuronal progenitor cell; qPCR, quantitative polymerase chain reaction.

In our previous work, we identified 48 circRNAs as significantly dysregulated in the BM44 region of the MSBB dataset in AD.^24^ To investigate the regional specificity of this dysregulation, we analyzed additional brain regions from the same dataset here. Notably, circPDE4B emerged as the only circRNA differentially expressed in AD across BM10, BM22, BM36, and BM44 (Figure 1A). In all four regions, circPDE4B expression was consistently reduced, whereas the expression of its corresponding linear transcript remained largely unchanged (Figure 1B). Importantly, we did not observe correlation between the expression levels of circPDE4B and its linear mRNA counterpart, PDE4B (Figure S4 in supporting information). We have previously validated circPDE4B reduced expression in independent cohort using qPCR^24^ and circPDE4B has also been shown to be reduced in AD by Dube et al.^23^ Together, the robust and region‑wide reduction of circPDE4B across datasets, coupled with prior independent reports, provided a strong rationale to investigate its functional role in AD. We next examined correlations with Braak stage, Clinical Dementia Rating (CDR), and Consortium to Establish a Registry for Alzheimer's Disease (CERAD) scores. circPDE4B showed modest negative correlations with Braak stage and CDR (r ≈ −0.3; Figure S5A in supporting information), but no association with CERAD. To dissect the contribution of Aβ and tau, we next examined circPDE4B expression using neuropathological and clinical stratification. We used CERAD scores to index Aβ plaque burden and Braak stage to index the regional distribution of neurofibrillary pathology, and restricted our analysis to individuals with minimal or no cognitive impairment (CDR < 1). First, we compared circPDE4B levels between cases with low versus high Aβ burden under a stringent tau restriction (Braak ≤ 2): *n* = 39; CERAD < 4, Braak ≤ 2, CDR < 1 versus *n* = 11; CERAD = 4, Braak ≤ 2, CDR < 1. This analysis revealed a trend toward decreased circPDE4B expression in individuals with high Aβ burden despite low Braak stage (Figure S5B). When we relaxed the tau restriction to Braak ≤ 3, comparisons between *n* = 56; CERAD < 4, Braak ≤ 3, CDR < 1 and *n* = 21; CERAD = 4, Braak ≤ 3, CDR < 1 no longer showed an appreciable difference in circPDE4B levels (Figure S5C). These results show that potential effects of amyloid burden on circPDE4B expression are evident, but only in the absence of significant tau pathology (up to Braak III). As tau pathology increases, the pathological and molecular heterogeneity might obscure the modest impact of Aβ on circPDE4B. However, given the relatively small sample size in this analysis, these findings should be considered preliminary and not conclusive.

To assess the relative abundance of circPDE4B compared to its linear counterpart, we quantified their expression in *post mortem* control brain samples (Figure 1C). Notably, circPDE4B exhibited a 3‐ to 4‐fold higher expression than the linear transcript, a trend that was also observed in iPSC‐derived neurons (Figure 1D). This pronounced enrichment relative to the linear transcript is rare among circRNA and suggests a strong functional role for circPDE4B. Interestingly, NPCs displayed comparable levels of circPDE4B and linear PDE4B expression (Figure 1E). However, upon differentiation of these progenitor cells into mature neurons, circPDE4B expression increased markedly, consistent with previous reports indicating global upregulation of circRNAs during neuronal maturation (Figure 1F).^39^, ^40^, ^41^ circPDE4B amplification was validated using divergent primers, Sanger sequencing, and RNase R treatment (ABM, Cat# E049; Figure S6 in supporting information). After RNase R digestion, qPCR analysis of circPDE4B levels showed only an ≈ 40% reduction, whereas linear PDE4B mRNA was 100% reduced, confirming the circular nature and resistance of circPDE4B to exonuclease degradation. Our findings collectively highlight that circPDE4B is a highly abundant circRNA that is a robustly dysregulated circRNA in AD. This unique expression profile strongly supports the need for its functional characterization to uncover its potential roles in disease mechanisms.

### 3.2 Downregulation of circPDE4B exhibits a strong translational response

**FIGURE 2:**
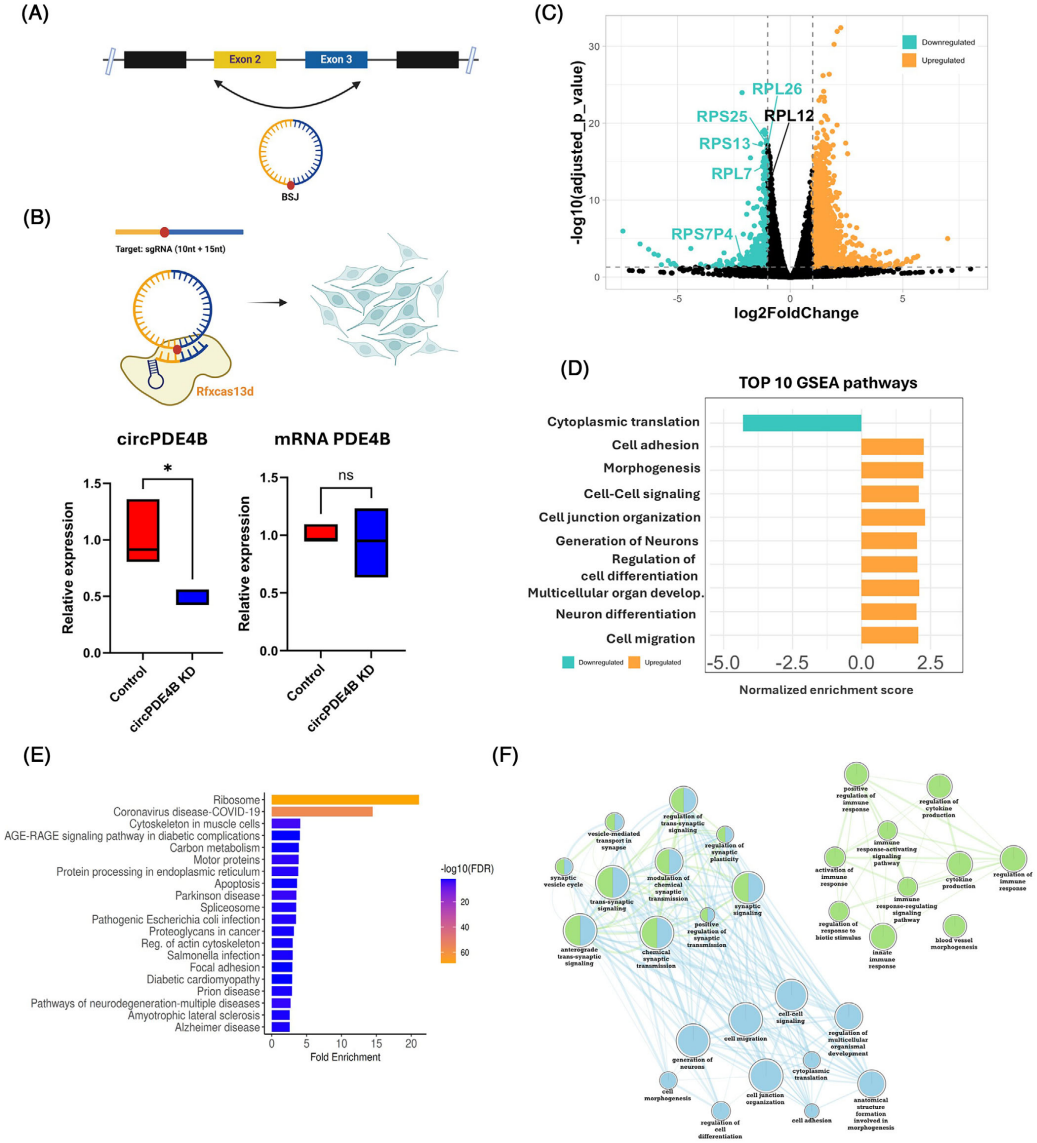
Downregulation of circPDE4B alters translational pathways. A, Schematic representation of circPDE4B showing back‐splicing between exons 2 and 3. B, qPCR validation of circPDE4B KD in NPCs using the RfxCas13d system (*n* = 3). C, Volcano plot showing differentially expressed genes after circPDE4B KD (log10 *p*adj > 1 and log2FC > 1). D, GSEA highlighting pathways altered by circPDE4B KD. E, Kyoto Encyclopedia of Genes and Genomes/Gene Ontology analysis identifying enrichment of neurodegenerative pathways. F, Enrichment map showing the top 10 overlapping pathways between circPDE4B KD in NPCs (green) and AD patient brain transcriptomes (Religious Orders Study and Memory and Aging Project; blue), along with the top 10 unique pathways for each dataset. Data are presented as mean ± standard error. *p* values are indicated; * *p* < 0.05 by unpaired Student *t* test. AD, Alzheimer's disease; GSEA, gene set enrichment analysis; KD, knockdown; mRNA, messenger RNA; NPC, neuronal progenitor cell; qPCR, quantitative polymerase chain reaction.

We first looked at the subcellular localization of circPDE4B in NPCs using BaseScope in situ hybridization and qPCR (Figure 1G,H), which revealed its localization predominantly in the cytoplasm. circPDE4B (hsa_circ_0008433) is generated by back‐splicing of exons 2 and 3 of the PDE4B gene (Figure 2A). The exon 2 encompasses the 5′ untranslated region as well as a portion of the coding sequence. The resulting circRNA is 351 base pairs in length. We went on to characterize the functional role of circPDE4B in NPCs, leveraging their homogeneity for greater experimental control than fully differentiated neurons. To investigate the functional effects of circPDE4B depletion, we used the RfxCas13d system to specifically knock down circPDE4B in NPCs, followed by bulk RNA sequencing to assess broader transcriptomic changes. We designed an asymmetric sgRNA targeting the unique backsplicing junction, spanning 10 nucleotides at the 3′ end and 20 nucleotides at the 5′ end (Table S2). This approach achieved efficient and selective KD of circPDE4B, confirmed by qPCR, with no impact on linear PDE4B mRNA (Figure 2B). A symmetric sgRNA spanning both ends of the junction was also tested, but proved less selective for the circular versus linear transcript. RNA‐seq analysis revealed significant transcriptomic changes (Figure 2C and Table S4 in supporting information), with gene set enrichment analysis (GSEA) showing an upregulation of genes involved in neuronal differentiation and a marked downregulation of genes related to translation (Figure 2D and Table S5 in supporting information). In line with this, KEGG Pathway analysis indicated that the differentially expressed genes were significantly enriched for ribosomal protein genes (Figure 2E). Figure S7 in supporting information shows a plot of transcript‐level changes for three representative ribosomal protein transcripts. In addition, we observed clear enrichment of AD and other neurodegenerative disorder KEGG terms (Figure 2E). Violin plots showing expression changes for selected transcripts from these AD‐related pathways are presented in Figure S8 in supporting information. To explore the disease relevance, we compared pathway enrichment results from circPDE4B KD to those derived from the Religious Orders Study and Memory and Aging Project (ROSMAP) AD datasets (Table S6 in supporting information). Strikingly, numerous pathways were commonly affected in both conditions. From these, we selected the top 20 overlapping pathways (Table S7 in supporting information) and the top 10 enriched pathways from each study for detailed analysis (Figure 2F). Notably, most of the overlapping pathways corresponded to neuronal and synaptic functions, including synaptic vesicle cycling, synaptic signaling, and chemical synaptic transmission (Figure 2F). Within these shared pathways, we identified a subset of transcripts that were upregulated in circPDE4B‐KD NPCs but downregulated in AD patient samples (Figure S9 in supporting information). This inverse pattern of gene expression suggests opposing regulatory effects of circPDE4B perturbation and AD pathology, highlighting potential context‐dependent roles for circPDE4B in neuronal function and disease.

**FIGURE 3:**
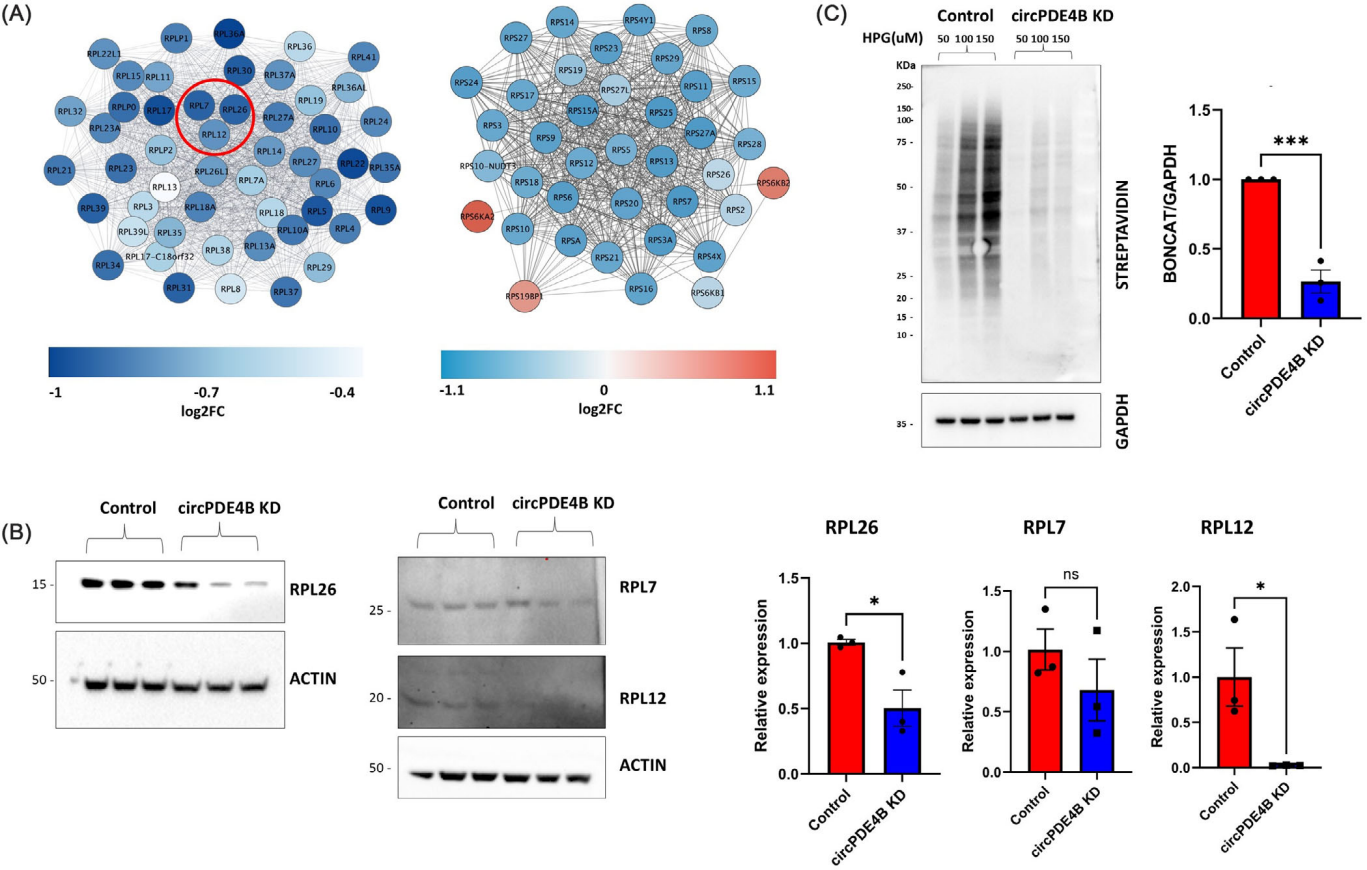
Downregulation of circPDE4B reduces translation. A, Network and expression analysis of ribosomal genes (RPS and RPL families) altered by circPDE4B KD. B, Immunoblot validation of selected ribosomal proteins (RPL26, RPL7, and RPL12; *n* = 3 biological replicate). C, BONCAT assay with immunoblot detection (streptavidin staining) showing reduced global translation upon circPDE4B KD (*n* = 3 biological replicate). Immunoblot data are normalized to GAPDH control. Data are presented as mean ± standard error. *p*‐values are indicated; *p < 0.05, **p < 0.01, ***p < 0.001, by unpaired Student *t* test. BONCAT, bio‐orthogonal non‐canonical amino acid tagging; GAPDH, glyceraldehyde 3‐phosphate dehydrogenase; HPG, homopropargylglycine; KD, knockdown.

The pronounced downregulation of translation‐related genes observed in the GSEA led us to hypothesize that circPDE4B plays a critical role in translational regulation. Specifically, analysis of the translation‐related gene network revealed substantial dysregulation of multiple ribosomal protein genes (Figure 3A). To validate these findings at the protein level, we examined the expression of RPL7, RPL12, and RPL26, and found that all three were significantly reduced in circPDE4B KD cells compared to controls (Figure 3B). To directly assess the impact on protein synthesis, we performed a bio‐orthogonal non‐canonical amino acid tagging (BONCAT) assay,^42^ which labels newly synthesized proteins in living cells. Consistent with our transcriptomic and immunoblot data, BONCAT analysis demonstrated a clear reduction in global protein translation upon circPDE4B KD (Figure 3C). Collectively, these results indicate that circPDE4B plays a critical role in regulating translational capacity in NPCs; this global translational downregulation shares similarity with a translational stress response.

### 3.3 Regulation of translation by circPDE4B is mediated by direct interaction with GEMIN5

**FIGURE 4:**
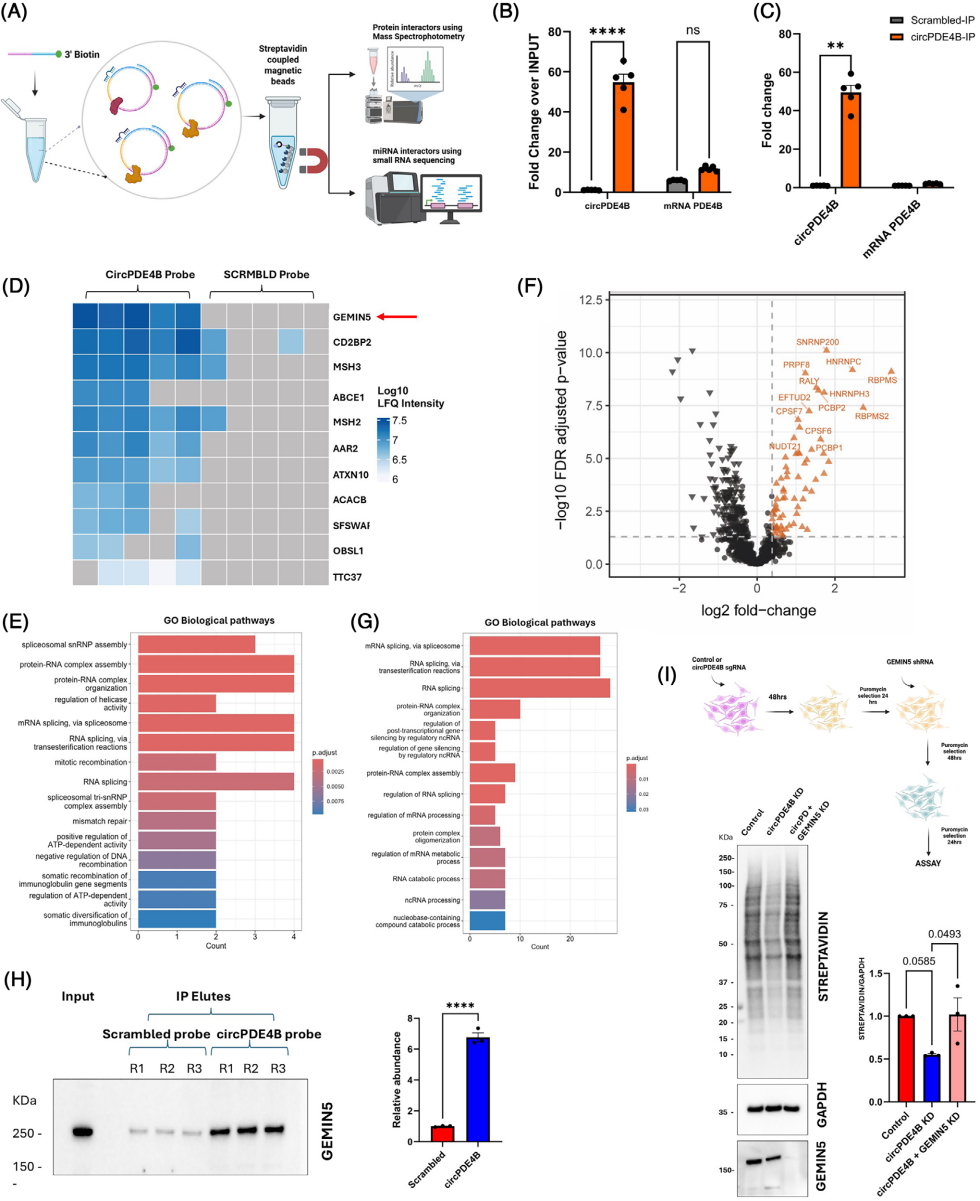
circPDE4B interacts with GEMIN5 to regulate translation. A, Schematic of circPDE4B RNA biotin/streptavidin precipitation in NPCs followed by mass spectrometry. B, qPCR validation of efficient pulldown, showing enrichment of circPDE4B over input with the circPDE4B probe compared to a scrambled probe, with no enrichment of linear PDE4B (*n* = 5 biological replicate). C, qPCR showing ≈ 40‐fold enrichment of circPDE4B in probe versus scrambled control. D, Heatmap of protein interactors exclusively identified in the circPDE4B probe pulldown. E, GO pathway analysis of 11 specific protein interactors. F, Volcano plot of enriched protein interactors (log2FC > 0.385, −log10FDR > 1.25). G, GO pathway analysis highlighting pathways associated with enriched protein interactors. H, Immunoblot validation of GEMIN5 enrichment in circPDE4B pulldown (*n* = 3). H, Schematic of circPDE4B KD followed by GEMIN5 KD, with BONCAT assay (streptavidin immunoblot) showing rescue of global translation in circPDE4B‐depleted cells (*n* = 3 biological replicate). Data are presented as mean ± standard error. *p* values are indicated; *p < 0.05, **p < 0.01, ***p < 0.001, by two‐way ANOVA for (B,C), unpaired Student *t* test for (H) and one‐way ANOVA for (I). ANOVA, analysis of variance; BONCAT, bio‐orthogonal non‐canonical amino acid tagging; GAPDH, glyceraldehyde 3‐phosphate dehydrogenase; GEMIN5, gem‐associated protein 5; GO, Gene Ontology; KD, knockdown; NPC, neuronal progenitor cell; qPCR, quantitative polymerase chain reaction.

The changes in transcripts coding for ribosomal proteins observed upon circPDE4B KD could reflect either direct or indirect regulatory effects. To explore the potential role of RBPs in these interactions, we performed affinity purification of circPDE4B to identify its associated RBPs. For interaction analysis, we designed a biotinylated probe targeting the unique backsplicing junction of circPDE4B, ensuring equal coverage of its 3′ and 5′ ends (Figure 4A). Using this probe, we achieved a 40‐fold enrichment of circPDE4B in the biotin/streptavidin‐precipitated fraction compared to scrambled control probe, and no enrichment for linear PDE4B confirming the specificity and efficiency of our approach (Figure 4B,C). The enriched complexes were subjected to mass spectrometry to identify RBPs associated with circPDE4B. Proteins detected in at least three circPDE4B and control replicates were used for initial statistical analysis, revealing 69 targets with increased pulldown by circPDE4B versus control (FDR‐adjusted *p* value < 0.05; fold‐change > 1.3; Figure 4F and Tables S8, S9 in supporting information). The enriched proteins fell into two groups. Figure 4D shows proteins that were present in the circPDE4B pulldown and absent in the negative control (pulldown with a scrambled probe sequence). These proteins comprised the strongest interactors but are not shown in the volcano plot (Figure 4F) because of the binary outcome. Figure 4F shows proteins that were enriched in the circPDE4B pulldown (but did show some binding to the negative control). Gene Ontology analysis revealed that majority of these interacting proteins are involved in RNA splicing and RNA–protein complex assembly, highlighting an important and striking connection between circPDE4B and key processes in RNA biology (Figure 4E,G). These included interactors PCBP1 and CPSF6, which we validated by immunoblot (Figure S10 in supporting information). Proteins identified consistently in circPDE4B purifications, but in less than three controls excluding them from differential statistics, showed additional key regulators (Figure 4D). Among these 11 identified proteins, functional enrichment analysis again emphasized roles in RNA processing and assembly (Figure 4G). Notably, the top rank specific interactor was GEMIN5, a critical component of the survival of motor neuron (SMN) complex.^43^ GEMIN5 is recognized for its multifaceted role in RNA metabolism, particularly in the assembly of small nuclear ribonucleoproteins and the regulation of translation.^44^, ^45^, ^46^ Given its prominent translational role, we proceeded to validate the circPDE4B–GEMIN5 interaction by immunoblot (Figure 4H).

The interaction between circPDE4B and GEMIN5 suggests a potential mechanism by which circPDE4B may regulate translation, possibly by modulating GEMIN5's availability or function within the translational machinery. Given that GEMIN5 acts as a translational repressor, we hypothesize that circPDE4B sequesters GEMIN5, thereby limiting its inhibitory effect on translation. Consequently, upon circPDE4B KD, increased levels of free GEMIN5 may lead to reduced global translation. To test this hypothesis, we performed circPDE4B KD followed by GEMIN5 depletion and assessed translation. Remarkably, GEMIN5 KD rescued the reduction in translation caused by circPDE4B depletion (Figure 4I). GEMIN5 KD alone in NPCs led to only a modest increase in protein synthesis, indicating that the rescue effect is specific to the context of circPDE4B loss (Figure S11 in supporting information). These results support a model in which circPDE4B modulates translational capacity in part by influencing GEMIN5, a multifunctional RNA‐binding protein known to interact with ribosomal subunits and regulate both global and selective translation through its distinct domains.

### 3.4 circPDE4B KD increases autophagy

**FIGURE 5:**
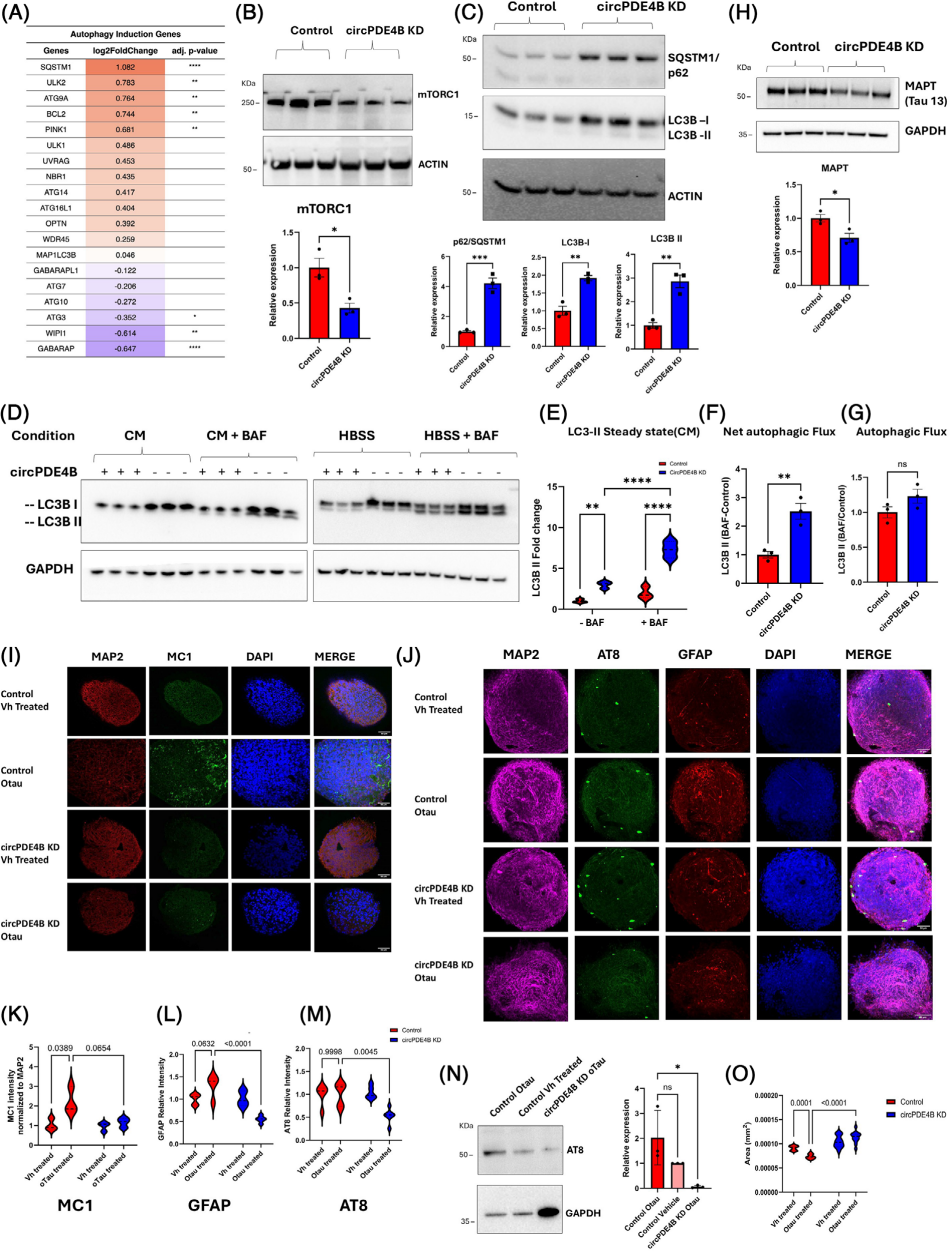
circPDE4B knockdown increases autophagic flux and MAPT pathology. A, Expression analysis of autophagy induction genes in circPDE4B KD cells. B, Immunoblot of mTOR protein levels in circPDE4B KD cells (*n* = 3 biological replicates). C, Immunoblots of p62 and LC3B‐I and II (*n* = 3 biological replicates). D, Autophagic flux assay: immunoblot detection of LC3 protein under autophagy induction and inhibition conditions (*n* = 3 biological replicates). E, Quantification of LC3B‐II levels under basal conditions with BAF treatment in both control and circPDE4B KD cells. F, Quantification of net autophagic flux calculated as the difference in LC3B‐II levels between BAF‐treated and control conditions. G, Quantification of autophagic flux determined by the ratio of LC3B‐II levels in BAF‐treated versus control samples. H, Immunoblot of MAPT expression (*n* = 3 biological replicates). I, Immunofluorescence of assembloids stained for MC1 (green), MAP2 (red), and DAPI (blue). J, Immunofluorescence of assembloids stained for AT8 (green), GFAP (red), MAP2 (pink), and DAPI (blue). K, Quantification of MC1 staining intensity (*n* = 3 assembloid, biological replicates). L, Quantification of GFAP staining intensity (*n* = 5 assembloid, biological replicates). M, Quantification of AT8 staining intensity (*n* = 5 assembloid, biological replicates). N, Immunoblot of AT8 expression (150 assembloids pooled per condition, *n* = 3 technical replicates). O, Quantification of assembloid size across conditions (*n* = 12 check). All immunoblot data were normalized to GAPDH or actin. Data are presented as mean ± standard error. *p* values are either indicated or defined as; *p < 0.05, **p < 0.01, ***p < 0.001, ****p < 0.0001 by two‐way analysis of variance. BAF bafilomycin; DAPI, 4′,6‐diamidino‐2‐phenylindole; GAPDH, glyceraldehyde 3‐phosphate dehydrogenase; GFAP, glial fibrillary acidic protein; KD, knockdown; MAPT, microtubule‐associated protein tau.

Our data indicate that circPDE4B can elicit a translational stress response through its interaction with GEMIN5. Because the translational stress response also regulates proteostasis pathways, we investigated other core pathways associated with translational stress, such as the mTOR–autophagy axis. Targeted examination of our circPDE4B KD transcriptomic data supported this observation, as we detected upregulation of several key autophagy‐related genes,^47^ including *SQSTM1*, *BCL2*, and *ULK2* (Figure 5A). It is well established that translational stress can lead to suppression of mTOR signaling, thereby activating autophagy as a compensatory mechanism.^48^ Immunoblot analysis revealed a significant reduction in mTOR protein levels after circPDE4B KD, consistent with the induction of a translational stress response (Figure 5B). At the protein level, we observed increased expression of autophagy markers SQSTM1 (p62) and both LC3B‐I (cytosolic) and LC3B‐II (in autophagosome membrane; Figure 5C). To directly assess autophagic flux, we performed LC3B immunostaining under both inhibition and induction of autophagy conditions. Blocking autophagosome–lysosome fusion using bafilomycin (BAF) A1 enables measurement of LC3B‐II to indicate the level of autophagosome formation.^49^ Under basal conditions in complete media, the observed increase in LC3‐II levels at steady state and the significant increase upon BAF treatment in circPDE4B KD compared to control indicates enhanced autophagosome formation (Figure 5D,E). In our study, we observed that circPDE4B KD resulted in an increase in net autophagic flux, as measured by the difference in LC3‐II levels between BAF‐treated and control samples (Figure 5F, G), indicating enhanced autophagosome turnover. Concurrently, there was a slight increase in the overall autophagic flux ratio (Control/BAF; Figure 5H). This suggests that while the absolute amount of autophagosomes being processed is elevated in the circPDE4B KD condition, the relative balance between autophagosome formation and degradation is only modestly shifted compared to control.^49^ Similar changes in LC3‐II are observed under the starvation‐mimicking HBSS condition, supporting the notion that circPDE4B modulation impacts autophagosome dynamics under different nutrient states (Figure 5D and Figure S12A in supporting information). It is noteworthy that although increased autophagic flux typically correlates with decreased p62 levels due to its degradation via selective autophagy, we observed elevated SQSTM1 levels in our system (Figure 5B). This apparent discrepancy may be explained by increased transcription of SQSTM1, as shown by elevated SQSTM1 transcript levels (Figure 5A). Alternatively, the increase in p62 could reflect non‐selective autophagy, in which the rate of p62 synthesis surpasses its degradation, leading to accumulation despite active autophagic processes. We also observed an increase in the autophagic regulator BAG3 (Figure S12B). Prior studies indicate that BAG3 upregulation and enhanced autophagic activity reduce MAPT levels.^50^, ^51^ Immunoblotting MAPT also demonstrates reduced MAPT levels upon circPDE4B KD (Figure 5H). However, whether the decrease in MAPT is driven primarily by increased autophagic degradation or secondary to reduced translation remains unclear, warranting further investigation.

### 3.5 circPDE4B KD mitigates MAPT pathology and astrocyte activation in an iPSC‐derived 3D tauopathy model

**FIGURE 6:**
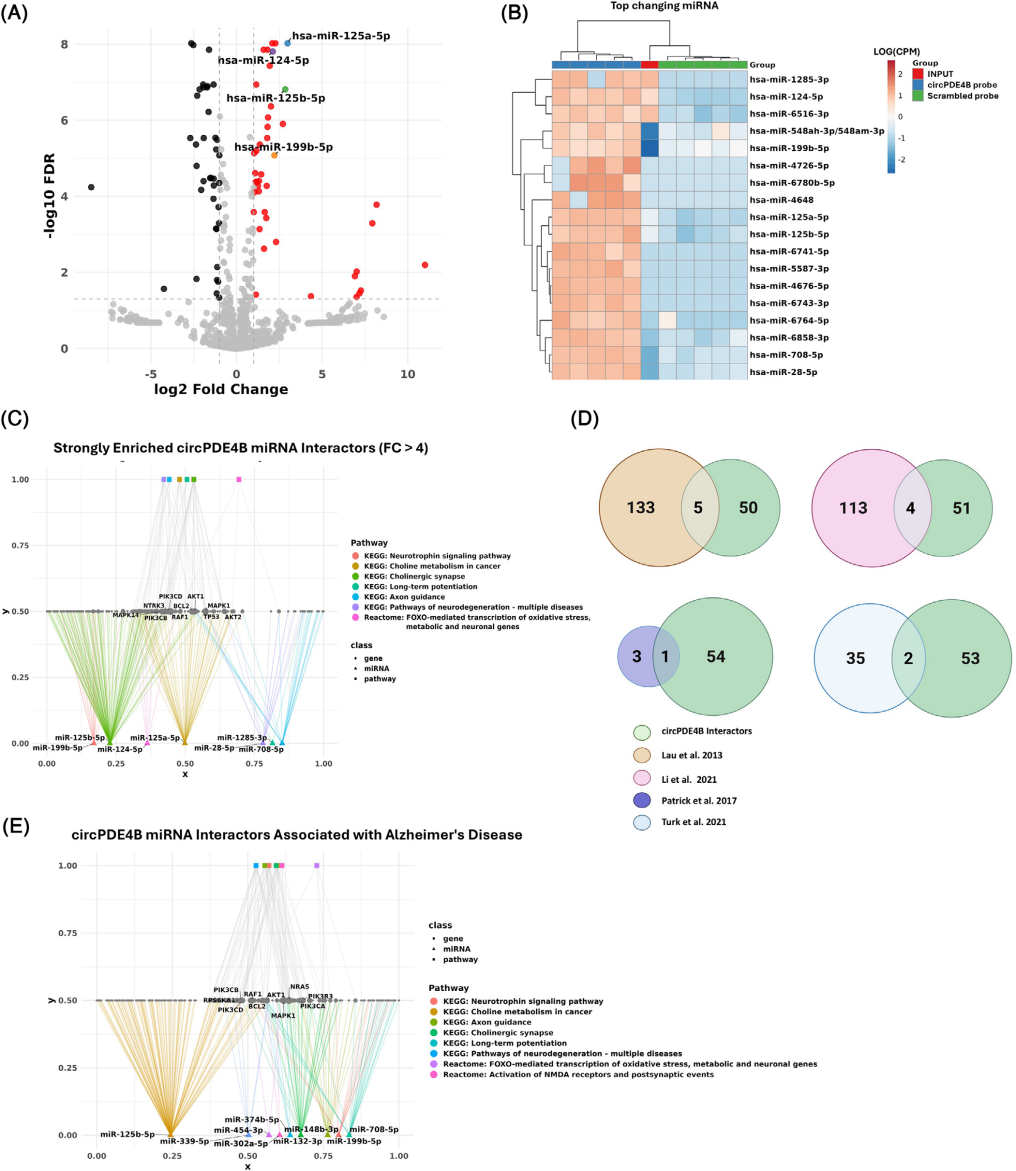
circPDE4B interacts with AD‐associated miRNAs. A, Volcano plot of miRNAs enriched in circPDE4B pulldown (log2FC > 1, −log10FDR > 1; checked against miRBase/miRTarBase; *n* = 5 biological replicate). B, Heatmap of enriched miRNAs with fold change > 4. C, Network analysis of circPDE4B‐interacting miRNAs, predicted target genes, and associated pathways, highlighting neurodegeneration‐related signaling. D, Overlap of circPDE4B‐interacting miRNAs with AD‐associated miRNAs identified in four independent datasets. E, Network analysis of 10 circPDE4B‐interacting miRNAs associated with AD, depicting convergent pathways of neurodegeneration. AD, Alzheimer's disease; miRNA, micro RNA.

The ability of circPDE4B KD to elicit a translational stress response, activate autophagy, and reduce total MAPT/tau led us to hypothesize that circPDE4B KD might be an effective tool to combat tauopathies. We investigated the effect of circPDE4B downregulation in our previously established iPSC‐derived tauopathy model.^38^, ^52^ In this system, exposure of iPSC‐derived neurons to oligomeric oTau followed by co‐culture with astrocytes induces pathological MAPT accumulation, marked by fibrillar MAPT detected with the MC1 antibody, alongside increased astrocytosis as indicated by glial fibrillary acidic protein expression. We performed circPDE4B KD in NPCs, which were then exposed to oTau and subsequently differentiated into neurons integrated with astrocytes into 3D assembloids. Assembloids generated from circPDE4B KD neurons exhibited a striking decrease in pathology compared to assembloids treated with vehicle, as determined by a reduction in pathological, misfolded MAPT (Figure 5I,K, measured with the MC1 antibody) and reduced astrocyte activation (Figure 6J,L). Phosphorylated MAPT levels also decreased (Figure 5J,M,N and Figure S13 in supporting information). Additionally, oligomeric MAPT exposure reduced overall assembloid size, an effect reversed by circPDE4B KD (Figure 5O). To further explore the underlying mechanism, we examined mTOR expression in circPDE4B–KD assembloids. Consistent with the results observed in NPCs (Figure S14A in supporting information), mTOR levels were significantly reduced after circPDE4B depletion. In contrast, we also observe circPDE4B expression itself was elevated in oTau‐treated assembloids compared to controls (Figure S14B). These results suggest that circPDE4B KD can elicit a strong protective action, reducing MAPT pathology and decreasing the astrocytic response in this human tauopathy model.

### 3.6 circPDE4B interacts with abundant miRNA linked to AD

We observed that circPDE4B interacts with several RNA‐binding proteins, and the functional effects we report are largely attributable to its interaction with GEMIN5. However, circRNAs are also known to act as competitive endogenous RNAs by interacting with and sequestering miRNAs.^11^ Given that circPDE4B is downregulated in AD and that its depletion in NPCs elicits a protective response, we sought to explore its association with miRNAs. Identifying miRNAs linked to circPDE4B could provide further insight into its functional roles and contributions to AD. We performed circPDE4B affinity purification followed by Ago2 immunoblotting, confirming interactions indicative of miRNA binding (Figure S15 in supporting information). Next, we sequenced the small‐RNAs associating with circPDE4B upon pull‐down; the sequencing identified 55 miRNAs with significant enrichment (fold change > 1.8, FDR < 0.05; Figure 6A and Table S10 in supporting information). From these, we selected 19 of the strongest interactors with a fold change > 4 (Figure 6B), including miR‐125a‐5p, miR‐125b‐5p, miR‐124‐5p, and miR‐199b‐5p, all known to play critical roles in neuronal development and function. Network analysis of these miRNAs revealed associations with pathways such as cholinergic synapses, axon guidance, and neurodegeneration (Figure 6C). To investigate disease relevance, we examined the dysregulation of these 55 miRNAs in AD using four independent datasets: Lau et al., analyzing hippocampus and prefrontal cortex cohorts;^53^ Li et al., assessing inferior frontal gyrus and superior temporal gyrus;^54^ Patrick et al., focusing on dorsolateral prefrontal cortex from ROSMAP cohorts;^55^ and Turk et al., compiling experimentally validated miRNA–target interactions associated with AD from miRTarBase and literature^56^ (Figure 6D and Table S11 in supporting information).

Among the 55 miRNAs interacting with circPDE4B, we identified 10 miRNAs commonly dysregulated in AD (Table S11). Network analysis highlighted pathways involved in synaptic function and neurodegeneration (Figure 6E). Notably, miR‐199b‐5p, detected at higher levels in cerebrospinal fluid of AD patients, promotes MAPT hyperphosphorylation by decreasing key regulators such as PIN1.^57^, ^58^ miR‐125b‐5p is upregulated in AD and drives MAPT hyperphosphorylation by downregulating the phosphatases DUSP6 and PPP1CA.^59^ Most importantly we observe circPDE4B interaction with miR‐132‐3p, a well‐established neuroprotective microRNA with multiple roles in AD pathology. MiR‐132‐3p is downregulated in AD brain regions, contributing to neurodegeneration by promoting neuronal apoptosis, MAPT pathology, and Aβ accumulation.^60^, ^61^ Overexpression of miR‐132‐3p ameliorates memory deficits, reduces amyloid burden, and protects neurons against MAPT‐related damage and excitotoxicity.^62^ Another miRNA interactor that we observe is miR‐339‐5p, which is also reduced in AD brain tissue and regulates BACE1 expression in human brain cells.^63^ To explore potential miRNA‐mediated effects, we examined the expression of validated mRNA targets of these 10 miRNAs in the circPDE4B KD RNA‐seq dataset. We found that a greater number of these target transcripts were upregulated than downregulated after circPDE4B KD (Table S12 in supporting information). This pattern does not support a classical “miRNA sponge” mechanism, in which increased availability of free miRNA would be expected to reduce target mRNA levels. These findings suggest that the translational changes and MAPT pathology rescue observed upon circPDE4B depletion in neuronal precursor cells are unlikely to be primarily driven by miRNA‐dependent regulation. Nonetheless, these miRNA interactions might have additional, context‐dependent implications for AD pathophysiology that warrant further investigation.

## 4 DISCUSSION

In this study, we provide a comprehensive functional characterization of circPDE4B, a circRNA associated with AD. Through analysis of an AD circular transcriptome dataset, we observed significant downregulation of circPDE4B in AD subjects. circPDE4B was first reported to be differentially expressed in AD by Dube et al.;^23^ further details on the association of circPDE4B with AD was provided by our subsequent publication.^24^ The gene *PDE4B* encodes a cyclic adenosine monophosphate (cAMP)–specific phosphodiesterase that regulates cellular signaling by hydrolyzing cAMP.^64^ This gene is critically involved in memory formation and regulation of neuroinflammation—two central processes in AD pathogenesis. Inhibition of *PDE4B* has demonstrated neuroprotective effects in an APP knock‐in mouse model, including improved spatial memory, restoration of brain glucose metabolism, and reduced inflammation.^65^, ^66^ Among the two circRNAs produced from the *PDE4B* gene locus, hsa_circ_0008433 is the most abundant and prominently dysregulated in AD, highlighting its potential biological importance. Interestingly, we found that circPDE4B is expressed at higher levels than its linear mRNA counterpart, suggesting that it may play a critical and distinct functional role beyond canonical *PDE4B* mRNA functions. These findings align with emerging evidence that brain‐enriched circRNAs act as key regulators of neurodegenerative disease pathology, often displaying expression changes independent of their linear host transcripts, indicative of distinct disease‐relevant regulatory mechanisms.^6^, ^16^ To date, circPDE4B has not been functionally characterized in neurobiology or neurodegenerative disease. Our work is the first to demonstrate a critical role for circPDE4B in NPCs, linking its downregulation to RNA translation, autophagic modulation, and MAPT pathology relevant to AD.

Upon KD of circPDE4B, we observed strong downregulation of translation‐related genes, particularly ribosomal proteins. In parallel, we detected upregulation of neuronal differentiation pathways, with increased mRNA levels of Tuj1 and NEFL, which were further confirmed by immunofluorescence and immunoblot analysis (Figure S16 in supporting information), respectively. Together, these gene expression changes suggest activation of a translational stress response, consistent with previous studies reporting that translational repression is coupled with enhanced differentiation programs under neural stress.^67^, ^68^, ^69^ Because circPDE4B influences multiple cellular pathways, it is possible that its function extends beyond those examined here and that it may participate in additional regulatory processes relevant to neural development and stress adaptation.

We hypothesize that circPDE4B downregulation in AD occurs as a compensatory rather than causal event. We observe opposing transcriptional changes for circPDE4B KD and AD. Reduced circPDE4B activates pathways that resemble a cellular stress response, lowering protein production and boosting autophagy. Such changes could help to maintain homeostasis in the face of stress caused by the pathophysiology of AD. Less circPDE4B might help protect cells facing injury or disease by adjusting energy use and supporting protein quality control.

In this context, we note that a correlation analysis revealed only a weak negative association between circPDE4B levels and Braak stage. This pattern suggests downregulation is not a primary disease driver but rather a secondary, potentially adaptive response triggered by accumulating MAPT pathology and clinical decline. Moreover, our additional analysis of amyloid‐positive cases spanning a range of Braak stages suggests that reduced circPDE4B expression may be detectable only at the earlier stages of tau pathology. To more definitively address this, future studies should examine circPDE4B expression stratified by AD stage and MAPT burden.

CircRNAs have been shown to activate transcription by recruiting RBPs to gene promoters;^17^ however, no direct evidence currently exists for circRNAs influencing translation. In our study, circPDE4B KD led to a reduction in translation, prompting investigation of its interacting RBPs. Among these, GEMIN5 emerged as a key downstream effector. GEMIN5 is a multifunctional RBP known to regulate translation at multiple levels. The N‐terminal domain of GEMIN5 directly associates with ribosomal proteins L3 and L4, thereby decreasing global translation by binding ribosomes and polysomes.^45^ Conversely, the C‐terminal domain enhances internal ribosome entry site (IRES)‐dependent selective translation by interacting with specific RNA motifs.^70^ GEMIN5 oligomerization is essential for its translation regulatory functions, affecting both cap‐dependent and cap‐independent mechanisms.^44^, ^46^ Increased GEMIN5 availability has been shown to negatively regulate overall translation, while its reduction impairs translation of certain critical mRNAs.^45^, ^71^

**FIGURE 7:**
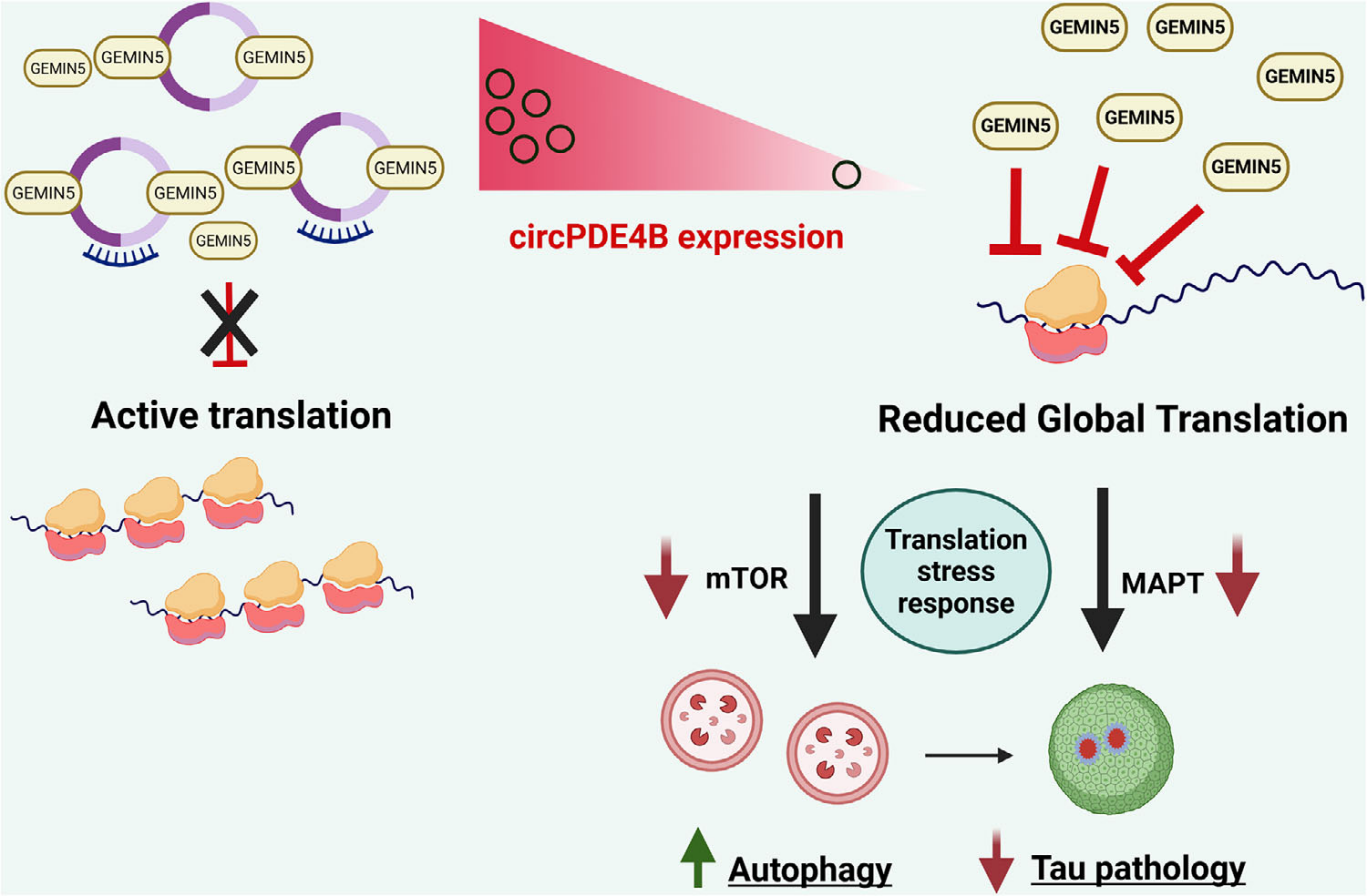
Schematic model depicting the biological role of circPDE4B. Interaction between circPDE4B and GEMIN5 regulates translation. High levels of circPDE4B sequester the translational inhibitor GEMIN5, thus increasing translation. Reducing circPDE4B allows higher levels of free GEMIN5, thus reducing translation. The reduced translation would increase cellular stress response pathways and activate autophagy, which further reduces levels of pathological MAPT. Illustration created using BioRender. GEMIN5, gem‐associated protein 5; MAPT, microtubule‐associated protein tau.

We propose a feedback loop model in which circPDE4B depletion increases the pool of free GEMIN5, which increases translational repression by GEMIN5 (Figure 7). We also observe reduced mTOR and MYC proteins (Figures 5, 7, and S17 in supporting information), which are two key regulators of ribosomal biogenesis. Modulation of these pathways appears to be sufficient to account for the inhibition of protein synthesis. The resulting biochemical changes also appear to stimulate the translational stress response and a global proteostasis response. Nonetheless, we cannot rule out that circPDE4B binds directly to motifs present on transcripts coding for ribosomal proteins. Future studies using circPDE4B affinity pulldown and sequencing of associated mRNAs will be necessary to determine whether circPDE4B also engages in direct regulatory interactions with rRNA and with ribosomal protein transcripts.

One of the key pathways stimulated during the translation stress response is autophagy, a process critical for the removal of pathological protein aggregates and maintenance of proteostasis. The ability of circPDE4B KD to reduce mTORC1 represents a clear pathway through which circPDE4B regulates autophagy.^72^ Several circRNAs have been shown to modulate autophagy via distinct mechanisms. For example, circST3GAL6 promotes autophagy by inhibiting the PI3K–Akt–mTOR pathway, while circPABPN1 reduces autophagy by blocking HuR binding to Atg16l1 mRNA.^73^ Additionally, circMUC16 facilitates autophagy by directly binding to the autophagy‐related protein ATG13, and circFOXO3 enhances autophagic flux by inhibiting the PI3K/AKT pathway.^74^ In our study, circPDE4B KD upregulated key autophagy induction genes and increased LC3B, supporting enhanced autophagic flux. Enhanced autophagy is known to facilitate the clearance of toxic protein aggregates such as MAPT/tau, which is central in AD pathology. Importantly, KD of circPDE4B reduced MAPT pathology in differentiated neurons in 3D assembloids, likely through increased autophagy, as evidenced by concurrent reductions in mTOR activity. The integrated mechanism through which circPDE4B KD provides protection could reflect synergistic interactions between increasing autophagy and increasing free GEMIN5 (thereby inhibiting translation and other functions linked to GEMIN5). Thus, these findings point to the actions of circPDE4B on multiple arms of proteostasis pathways, while supporting the hypothesis that circPDE4B is a strong regulator of proteostasis.

Although circPDE4B expression is decreased in AD patients and acute experimental KD shows protective effects, chronic reductions likely reflect complex disease dynamics involving multiple dysregulated pathways and compensatory mechanisms. Such compensatory reductions in RNAs or proteins are increasingly recognized in AD and other neurodegenerative diseases.^75^, ^76^, ^77^, ^78^ AD is multifactorial and dynamic, with gene expression changes influenced by diverse cell types and disease stages in *post mortem* brain tissue. In our tauopathy assembloid model, however, circPDE4B expression is increased after oTau exposure, consistent with its protective role in this acute experimental context. This apparent discrepancy highlights the distinction between chronic disease states and acute experimental systems, as models such as iPSC‐derived neurons may only partially capture the complexity of chronic conditions like AD.

Interactions between circRNAs and miRNAs are well documented and the current study fits this pattern. We found that circPDE4B interacts with several AD‐associated miRNAs, most notably miR‐132‐3p, which is significantly downregulated in AD brain tissue, plasma, and extracellular vesicles, and is recognized for its neuroprotective role in the early stages of the disease.^79^, ^80^ Loss of miR‐132‐3p leads to increased MAPT phosphorylation and aggregation, neuronal apoptosis, and heightened inflammation, while restoring its levels rescues these pathological processes in preclinical AD models.^60^, ^61^, ^62^, ^81^ The interaction between circPDE4B and miR‐132‐3p presents an intriguing pathway that could also impact the pathophysiology of AD, but further investigation is needed to elucidate the mechanisms and resulting phenotypes of this putative interaction.

Circular RNAs have been reported to encode small peptides in certain contexts. Given that circPDE4B contains a 5′ UTR fragment and that GEMIN5 is known to regulate IRES‐dependent translation, we examined the coding potential of circPDE4B. Analysis using circAtlas 3.0 identified three predicted IRES motifs and three potential open reading frames within the circPDE4B sequence. The predicted IRES elements had scores of ≈ 0.5, suggesting possible IRES activity based on computational criteria. The interaction between circPDE4B and GEMIN5 further supports the possibility of context‐dependent translation. Future studies using GEMIN5 iCLIP to map binding sites or circPDE4B overexpression constructs tagged with FLAG could determine whether circPDE4B gives rise to detectable peptide products.

In conclusion, our results indicate that circPDE4B is a powerful regulator of cellular stress responses. Its downregulation appears to activate stress‐ and autophagy‐related pathways, which may facilitate the reduction of pathological protein aggregation, a key therapeutic aim in neurodegeneration. Importantly, we discovered a strong regulatory axis that is mediated by GEMIN5, itself a strong regulator of neuronal function. The broad significance of circRNAs in neurodegenerative processes is only beginning to be understood. Our current study of circPDE4B reveals a mechanism of action that is completely different than its cognate linear transcript and acts via major regulators of proteostasis, including the RNA binding protein GEMIN5, and the signaling protein mTOR. These findings add to a growing body of work showing circRNA‐mediated control of stress adaptation and aggregate clearance, ultimately helping to unlock their therapeutic potential in neurobiology.

## 5 LIMITATIONS

This study has several important limitations. The heterogeneity of ADRDs is an important area of investigation that is not addressed in this article. The study does not include AD patient‐derived iPSCs, which would better test the generalizability of our findings and clarify whether acute protective effects persist in disease‐relevant genetic backgrounds. Multiple potential regulatory mechanisms remain to be explored. circPDE4B could regulate transcripts coding for ribosomal proteins by direct binding. The role of miRNAs in circPDE4B function needs more study. Finally, we have yet to determine whether circPDE4B produces peptides and how such peptides might act; the strong abundance of circPDE4B suggests that such putative peptides might be similarly abundant. The regulatory mechanisms linking circPDE4B KD to the pathophysiology of ADRD provides an area ripe for further investigation.

## CONFLICT OF INTEREST STATEMENT

B.W. is co‐founder and chief scientific officer for Aquinnah Pharmaceuticals Inc. A.E. is co‐founder and a scientific advisor to Prisma Therapeutics Inc. All other authors declare no relevant conflicts of interest pertaining to this article. Author disclosures are available in the Supporting Information.

## CONSENT STATEMENT

Human subject consent was not required for this study.

## CONSENT FOR PUBLICATION

All authors have approved the contents of this manuscript and provided consent for publication.

## Supporting information




Supporting Information

Supporting Information

Supporting Information

## Data Availability

RNA‐seq data have been deposited in the Gene Expression Omnibus (GEO) under accession GSE311546 and are publicly available as of the date of publication. The proteomics data have been deposited at ProteomeXchange with identifier PXD070615. All other data associated with this study are presented in the main text or supporting information. Any additional information required to reanalyze the data reported in this paper is available from the lead contact upon request. This study did not generate new unique reagents. The plasmids generated in this study can be obtained from lead contact with a completed materials transfer agreement.
