# Supplementary material for: circPDE4B downregulation triggers GEMIN5‑dependent translational stress response and autophagy to reduce MAPT pathology
Source: Alzheimers Dement. 2026 May 4;22(5):e71436. doi: 10.1002/alz.71436 (PMC13137278; doi:10.1002/alz.71436)
Supplement: Supplementary file 1 — Supporting Information [file ALZ-22-e71436-s003.docx]

**
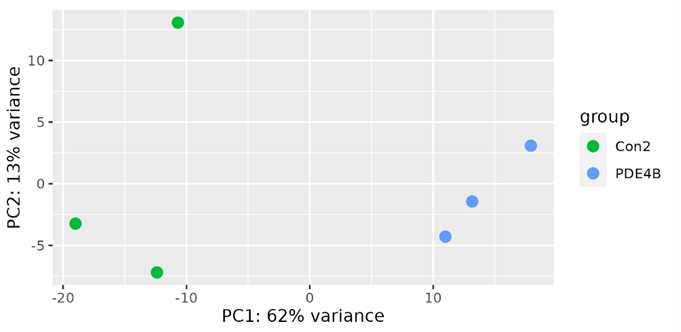
**

circPDE4B KD

Control

**Supplementary Figure 1.** Principal component analysis (PCA) of RNA-seq data. PCA plot of transcriptomic profiles from circPDE4B knockdown (KD) and control cells, showing separation between experimental groups

**
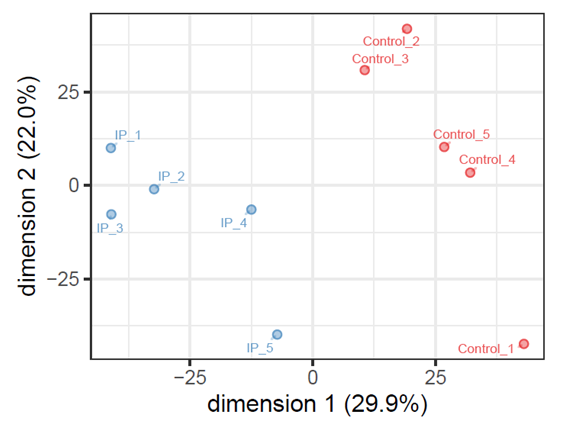
**

**Supplement Figure 2.** PCA plot of proteomics profiles from circPDE4B Affinity-pulldown(AP) and control, showing separation between experimental groups. Red: Control AP, Blue: circPDE4B AP


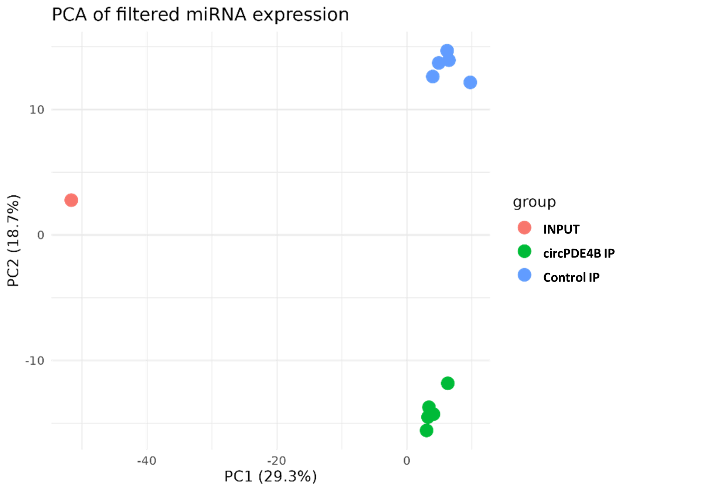


**Supplement figure 3.** PCA plot of miRNA expression profiles from circPDE4B Affinity-pulldown and control, showing separation between experimental groups.


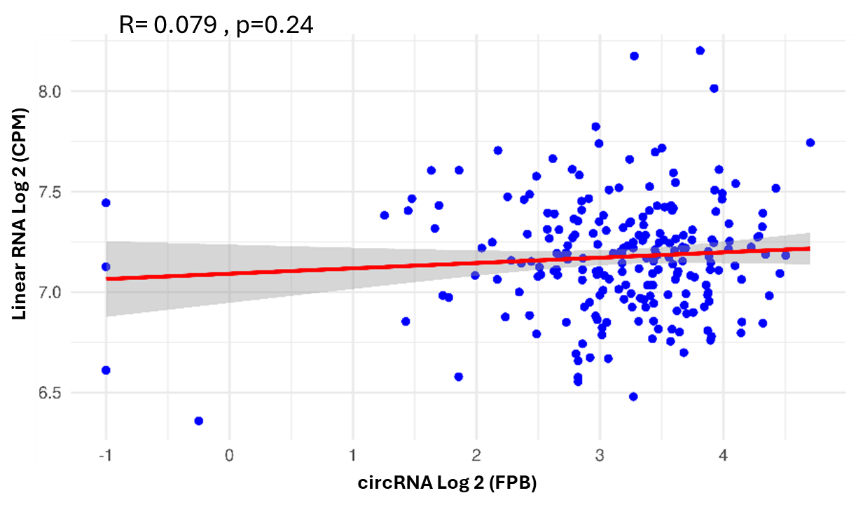


**Supplementary Figure 4.** Correlation between circPDE4B and linear PDE4B expression. Scatter plot depicting the correlation between circPDE4B and PDE4B mRNA expression in MSBB samples from AD patients and controls

**A**

**
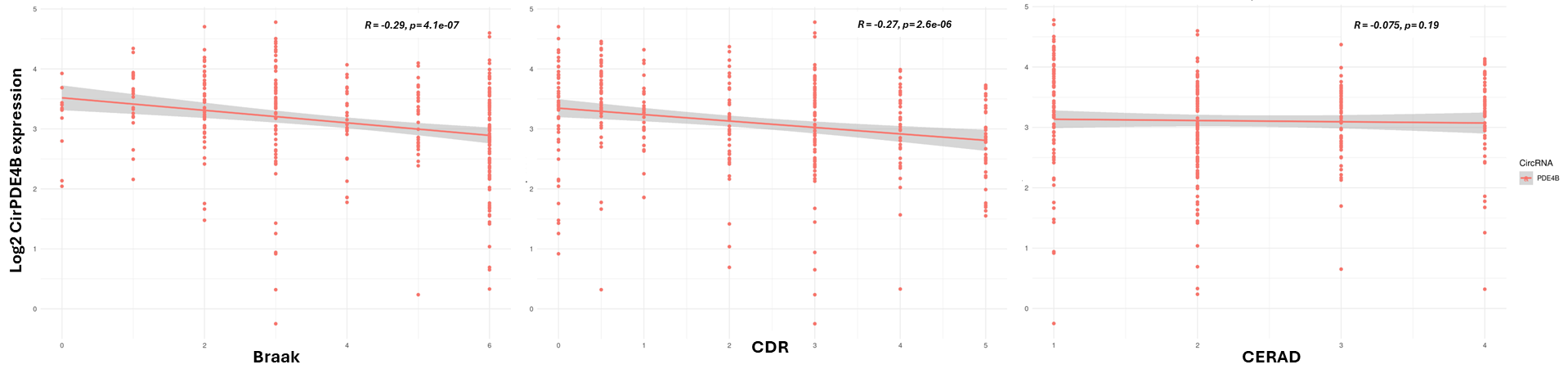
**


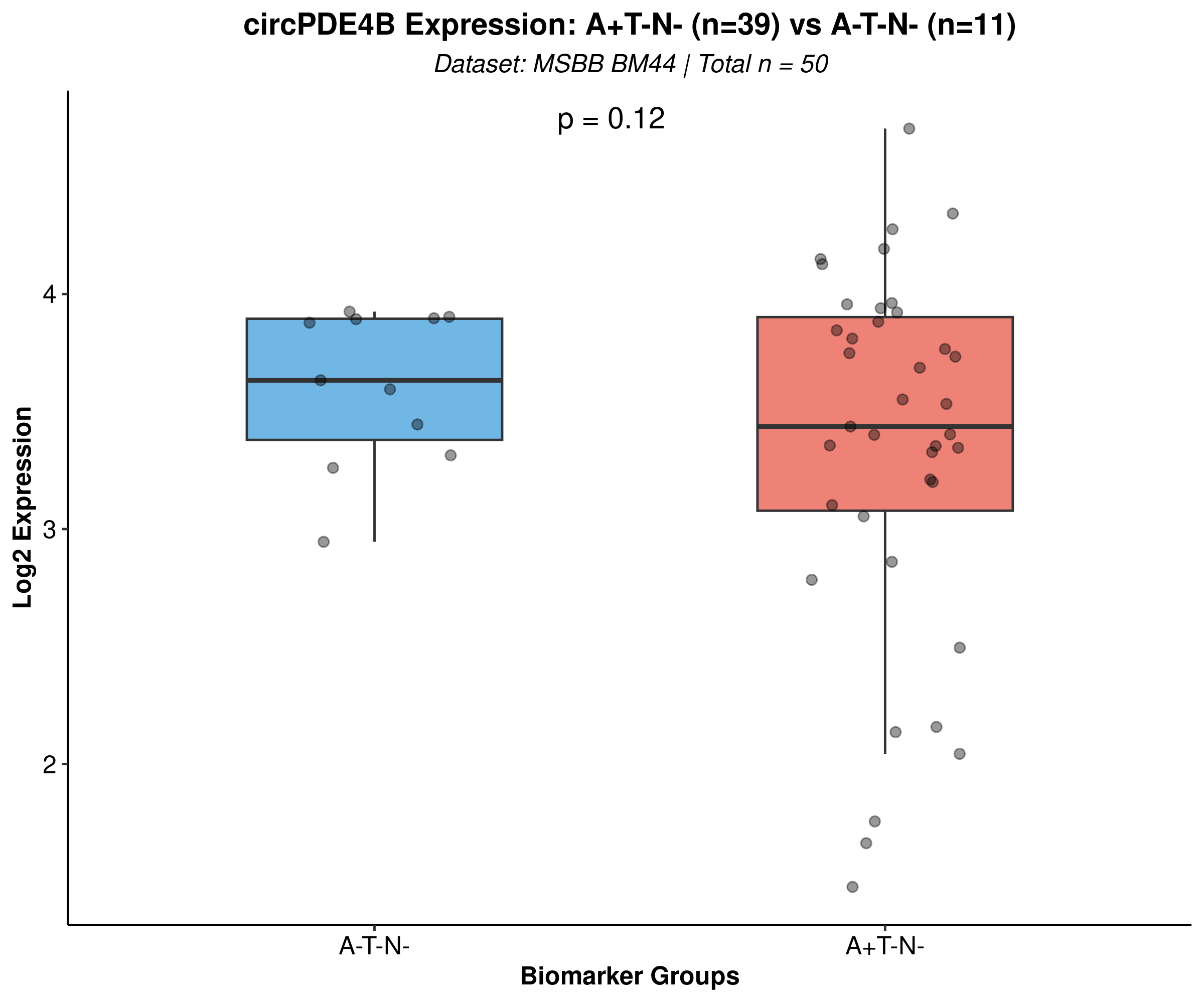

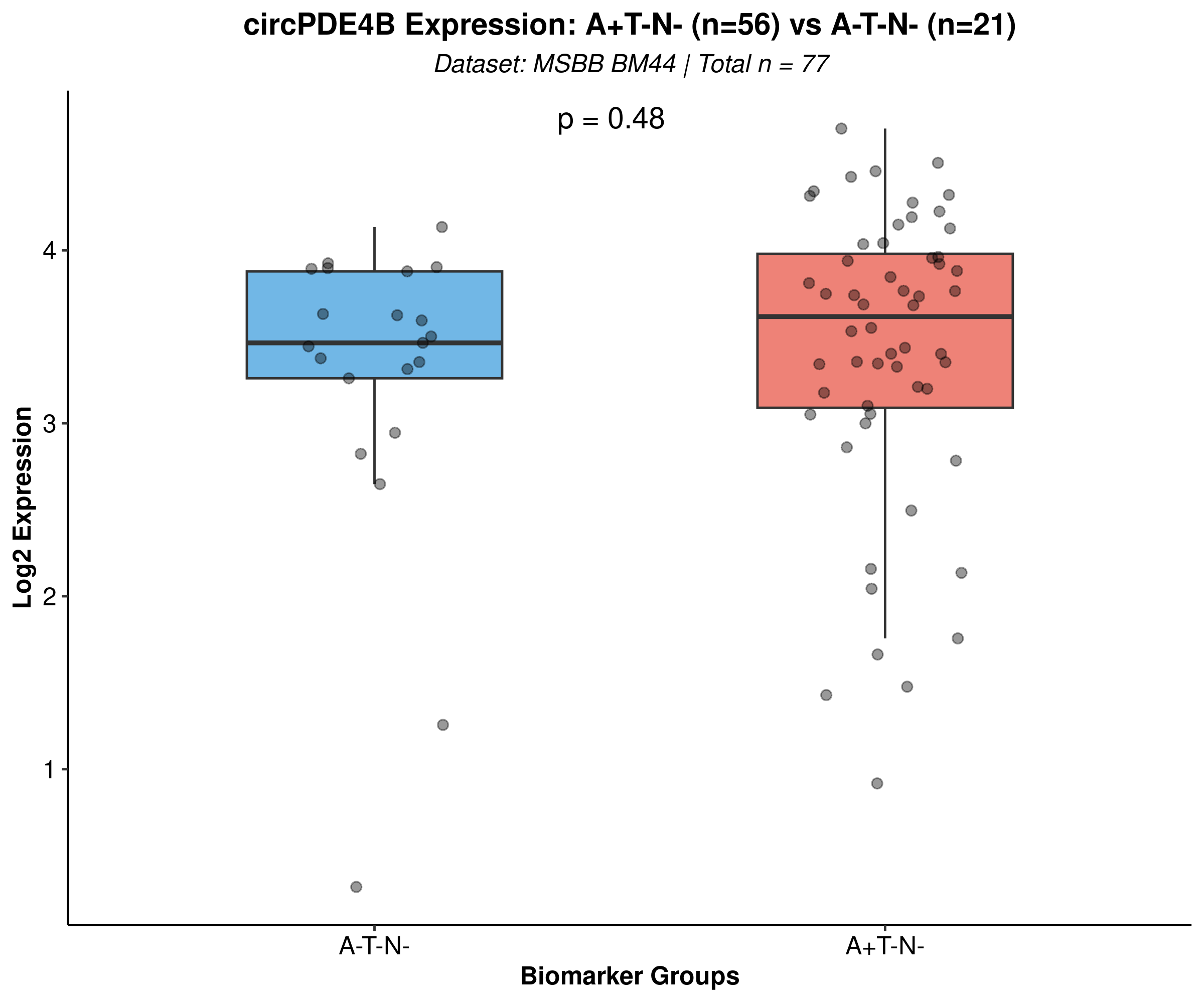
**B C**

**Supplementary Figure 5.** circPDE4B expression with AD pathology measures. (A) Scatter plots showing circPDE4B expression versus (A) Braak stage, Clinical Dementia Rating (CDR), and CERAD score in MSBB cohort samples from AD patients. (B) Log₂-transformed circPDE4B expression (x-axis) comparing A+T−N− cases and A−T−N− controls using a stringent definition of T− (Braak ≤ 2). A+T−N− cases (n = 39): CERAD < 4, Braak ≤ 2, CDR < 1. A−T−N− controls (n = 11): CERAD = 4, Braak ≤ 2, CDR < 1. (C) Log₂-transformed circPDE4B expression using a more permissive definition of T− (Braak ≤ 3). A+T−N− cases (n = 56): CERAD < 4, Braak ≤ 3, CDR < 1. A−T−N− controls (n = 21): CERAD = 4, Braak ≤ 3, CDR < 1.

**A**

**
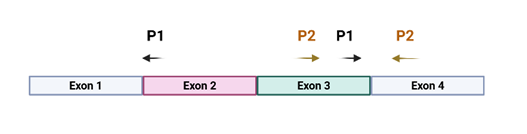
**

**B C**

**
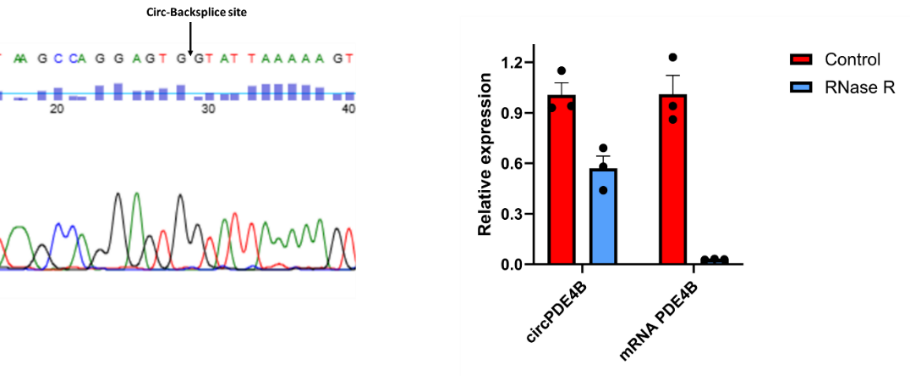
**

**Supplementary Figure 6.** Validation of circPDE4B backsplice junction and RNase R resistance. (A) Schematic showing the location of divergent primers (P1, black) designed to amplify circPDE4B spanning the back-splice junction between exon 2 and exon 3, and convergent primers (P2, yellow) designed to detect the linear PDE4B mRNA. (B) Sanger sequencing of PCR product amplified with divergent primers spanning the circPDE4B backsplice junction. Sanger chromatogram with the backsplice junction indicated by a vertical line. (C) RNase R resistance assay for circPDE4B. Left: qRT-PCR showing relative abundance of circPDE4B and linear PDE4B mRNA after RNase R treatment (+RNase R) versus control treatment (-RNase R). circPDE4B is resistant to RNase R (little reduction), while linear PDE4B is substantially reduced after treatment. Data represent n = 3 biological replicates.


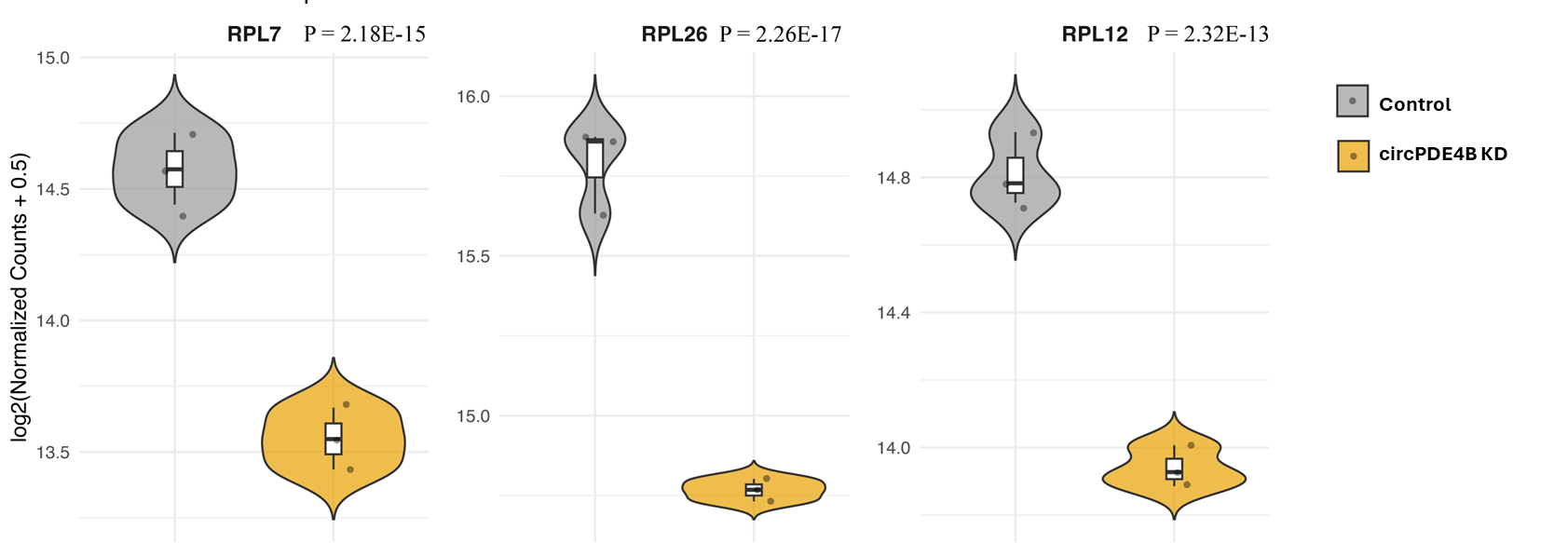


**Supplement Figure 7:** Violin plots showing transcript expression levels of ribosomal protein genes RPL7, RPL26, and RPL12 from the RNA-seq dataset across experimental conditions. Expression values are shown as log₂(normalized counts + 0.5). Each plot represents distributions derived from n = 3 biological replicates per condition.


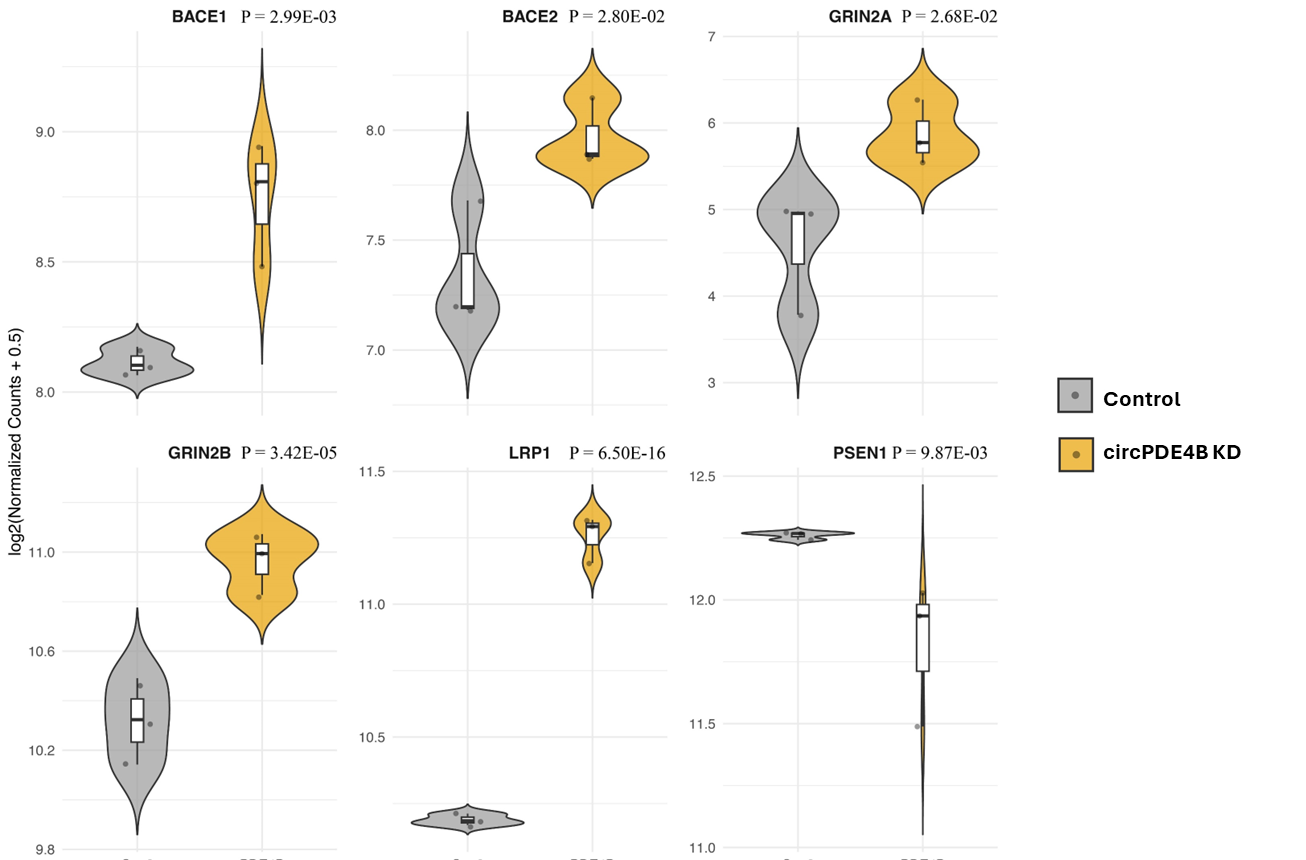


**Supplement Figure 8:** Violin plots showing transcript expression levels of selected genes from KEGG Alzheimer’s disease pathway enrichment in the RNA-seq dataset across experimental conditions. Expression values are shown as log₂(normalized counts + 0.5). Each plot represents distributions derived from n = 3 biological replicates per condition.

**A B**


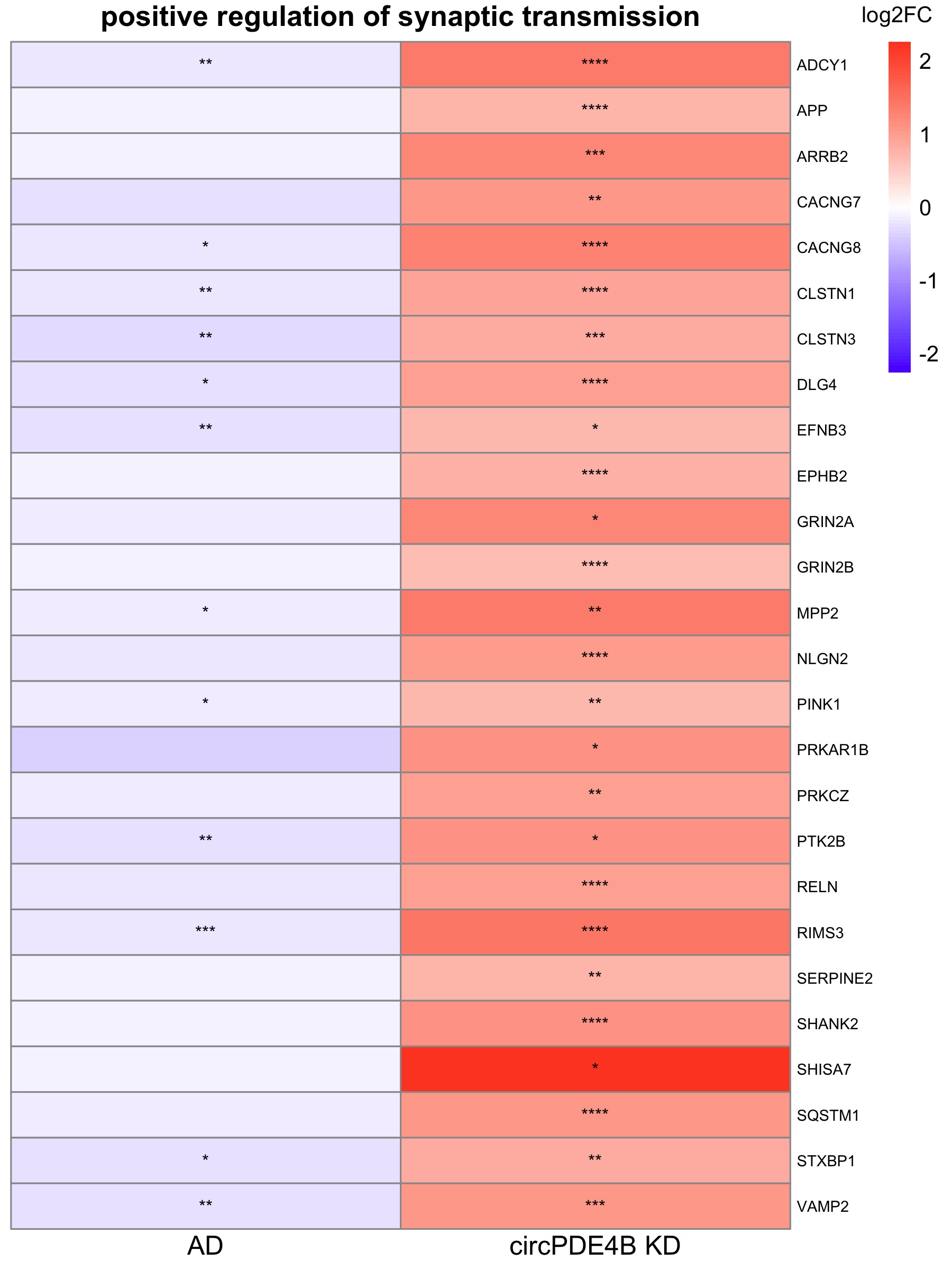

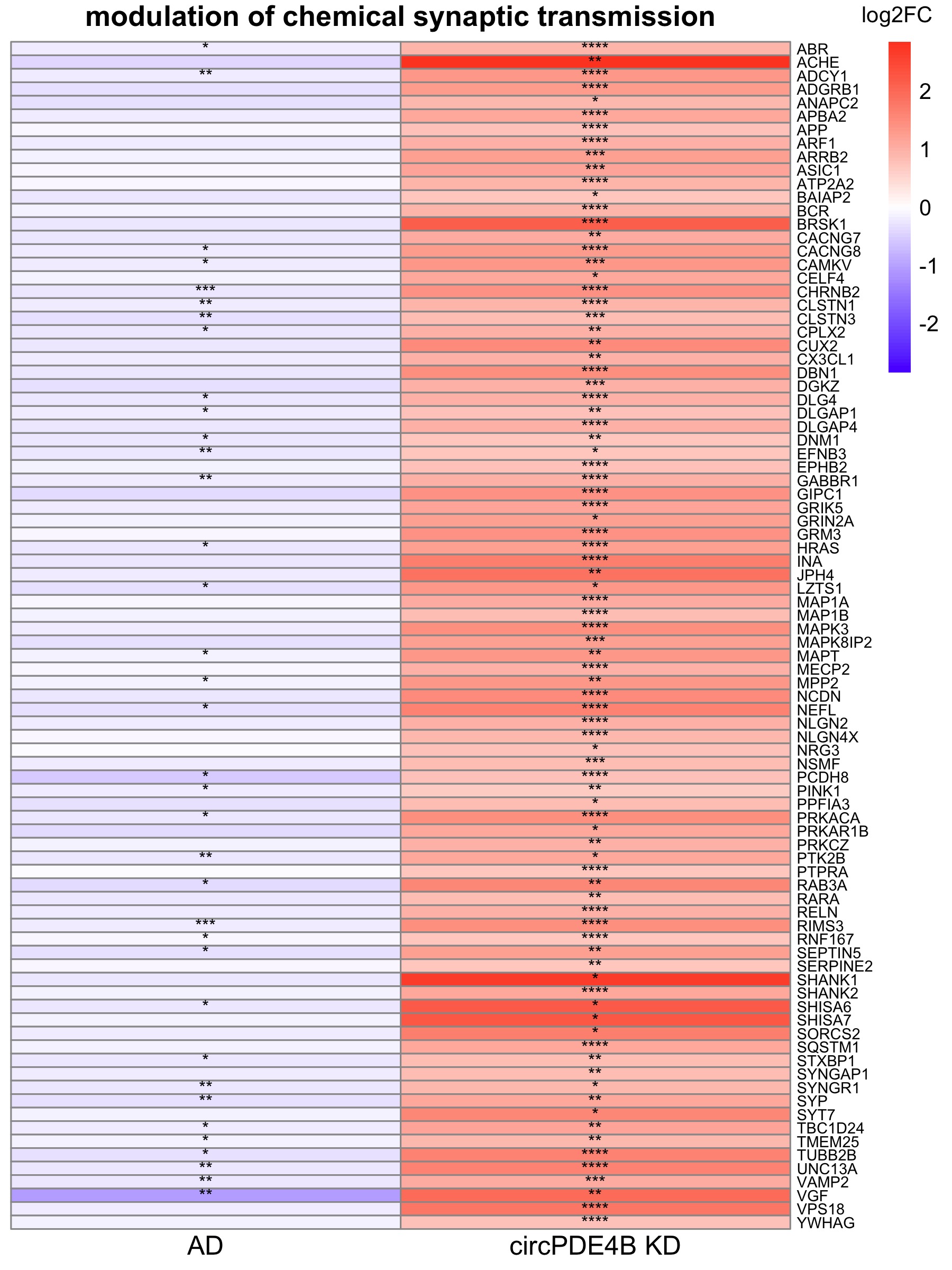


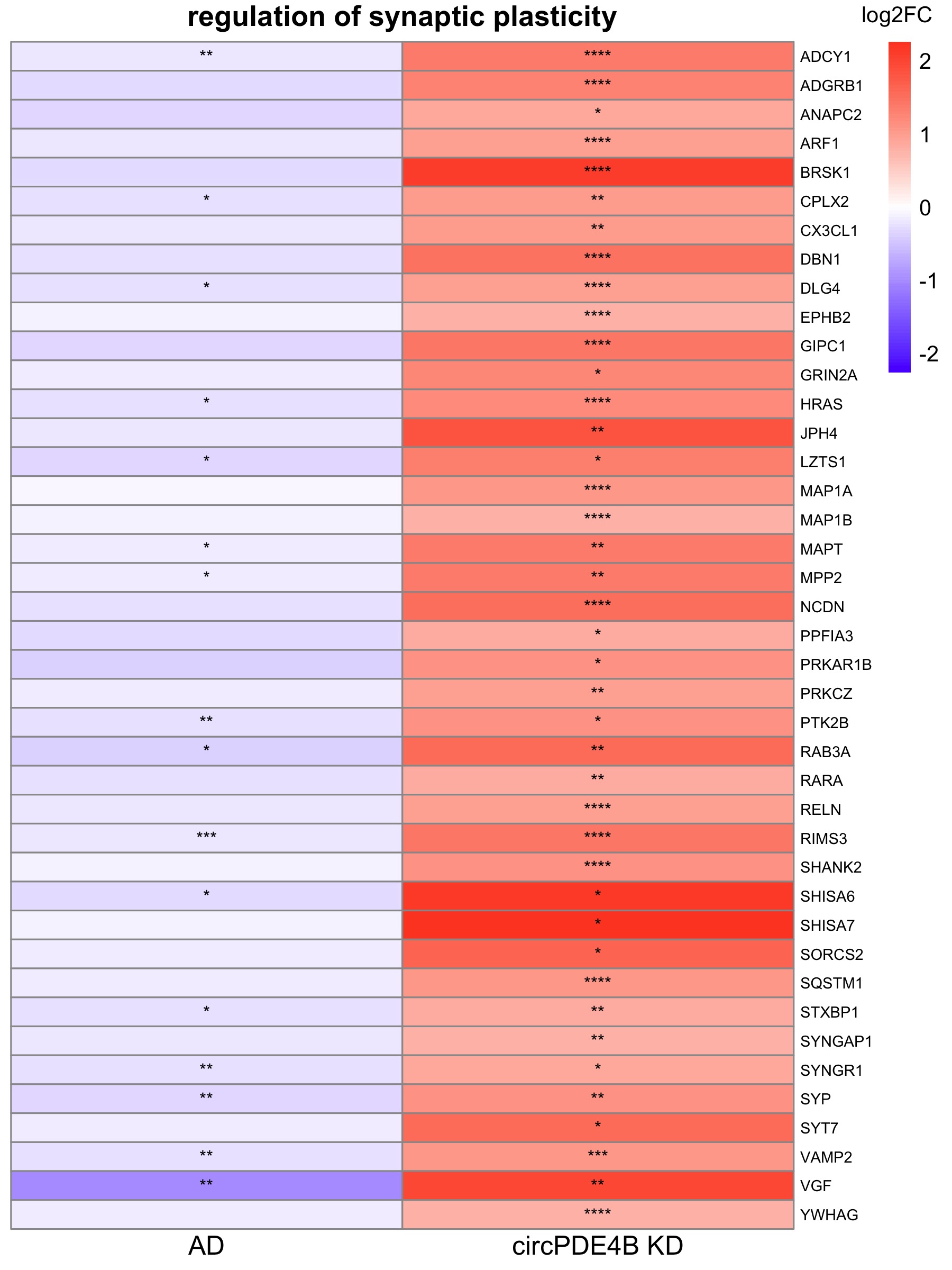
**C**

**Supplement Figure 9: Heat maps showing log fold-changes of overlapping transcripts in AD brains and circPDE4B KD** across top three shared pathways, (A) positive regulation of synapse transmission, (B) modulation of chemical synapse transmission, and (C) regulation of synaptic plasticity. Heat maps illustrate both the extent of gene-level overlap between conditions and the reversal in directionality of expression changes between AD and circPDE4B-KD.

**A**


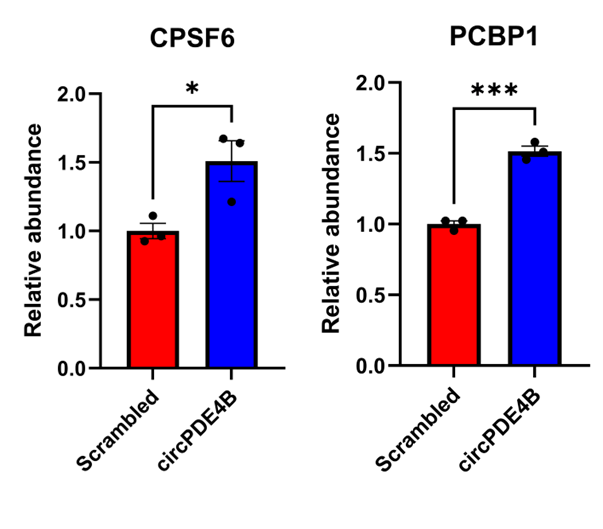
 **CPSF6 PCBP1**
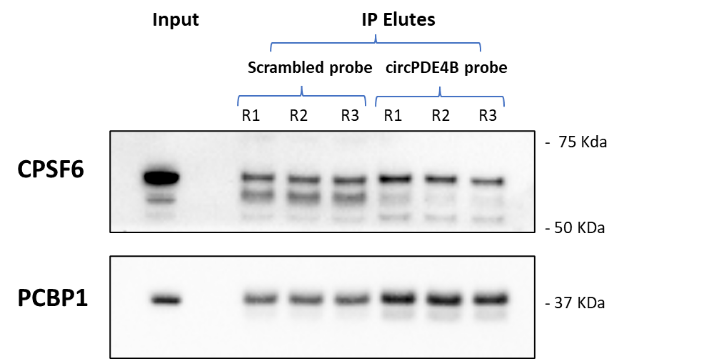


**B**

**
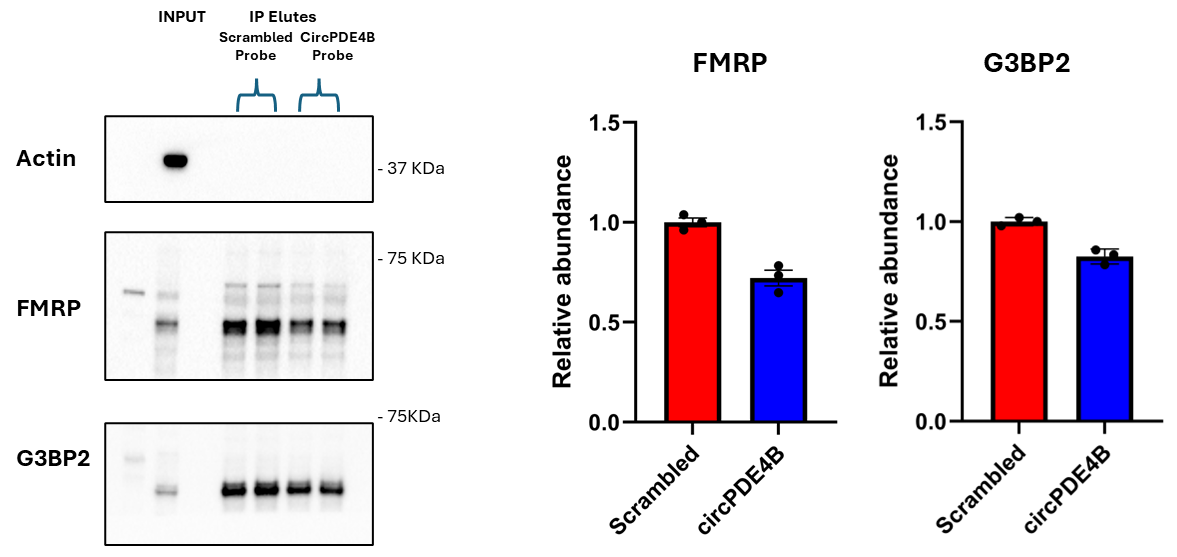
**

**Supplement Figure 10.** Immunoblot validation: (A) Additional proteins: CPSF6 and PCBP1 show enrichment in circPDE4B pulldown (n = 3). (B) Negative controls: Actin shows no enrichment in either elute; FMRP shows greater enrichment in scrambled control; G3BP2 shows comparable enrichment in scrambled and circPDE4B pulldown samples. (n=3). Data are presented as mean ± SE. P-values are indicated; *p < 0.05, **p < 0.01, ***p < 0.001 by unpaired student-t test.

**
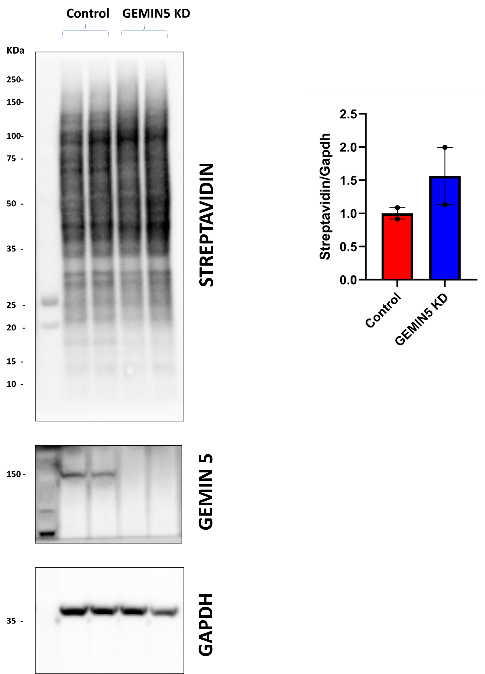
**

**Supplement Figure 11.** BONCAT assay with immunoblot detection (streptavidin staining) showing slight increase in global translation upon GEMIN5 KD knockdown on NPCs (n = 2 biological replicate). Immunoblot data is normalized to GAPDH control. Data are presented as mean ± SE

**A B**


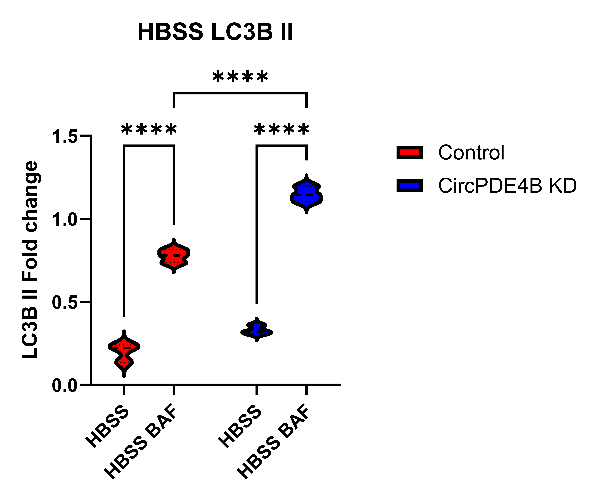
**
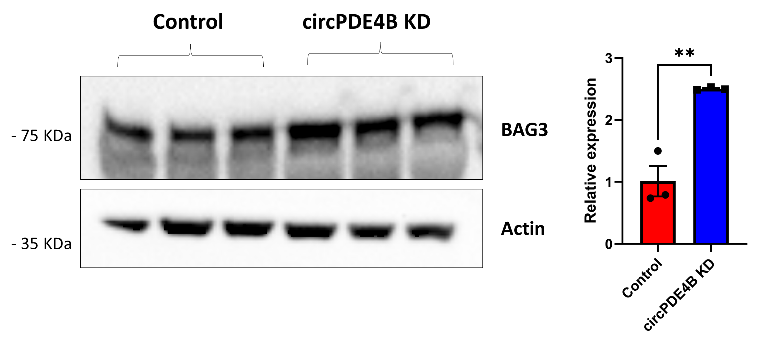
**

**Supplement figure 12.** (A) Quantification of LC3B-II levels under starvation conditions (HBSS) with BAF treatment in both Control and circPDE4B KD cells. Data are presented as mean ± SE. P-values are indicated***p < 0.0001, by Two-way ANOVA. (B) Immunoblot showing BAG3 increase in circPDE4B downregulated cells (n=3). Data are presented as mean ± SE. P-values are indicated; **p < 0.01, by unpaired student-t test.

**
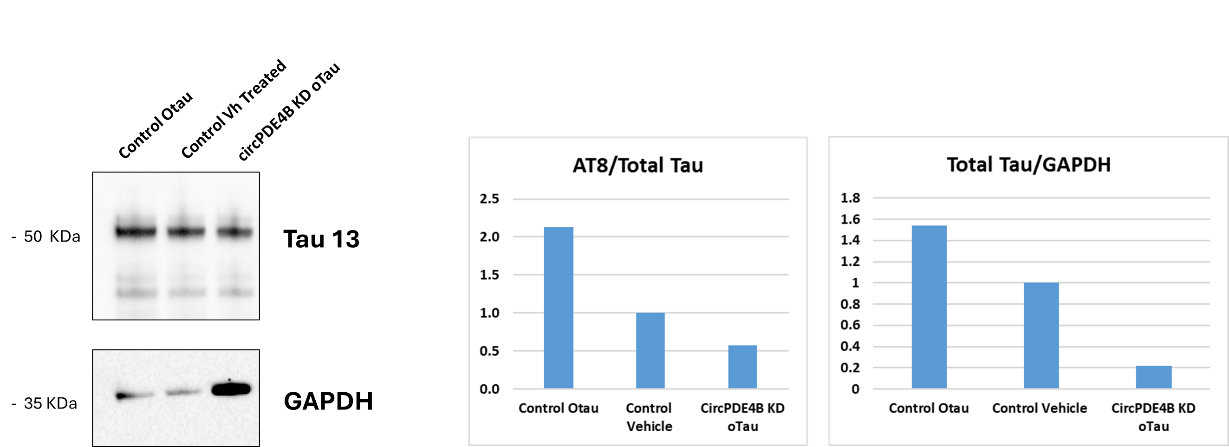
**

**Supplement Figure 13.** Immunoblot analysis of total tau (Tau13) in 3D assembloids model of tauopathy under different conditions, with quantification of phospho-tau (AT8) normalized to total Tau13**.** Data on y-axis is band density normalized to GAPDH. n=1

**
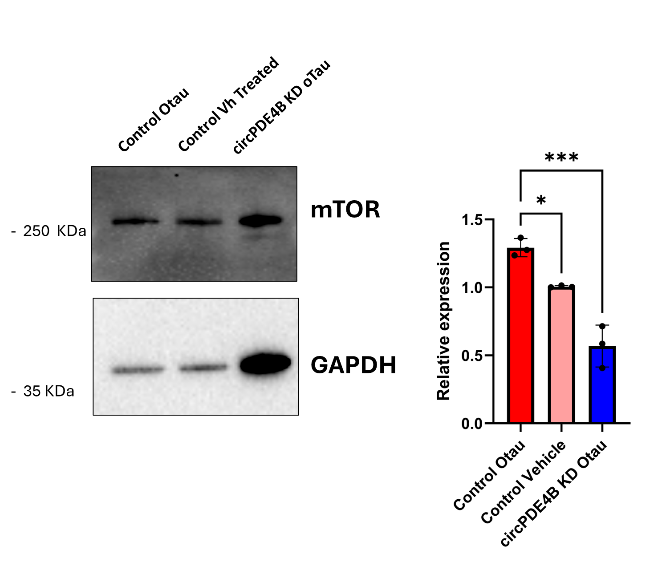
A B**

**CircPDE4B**


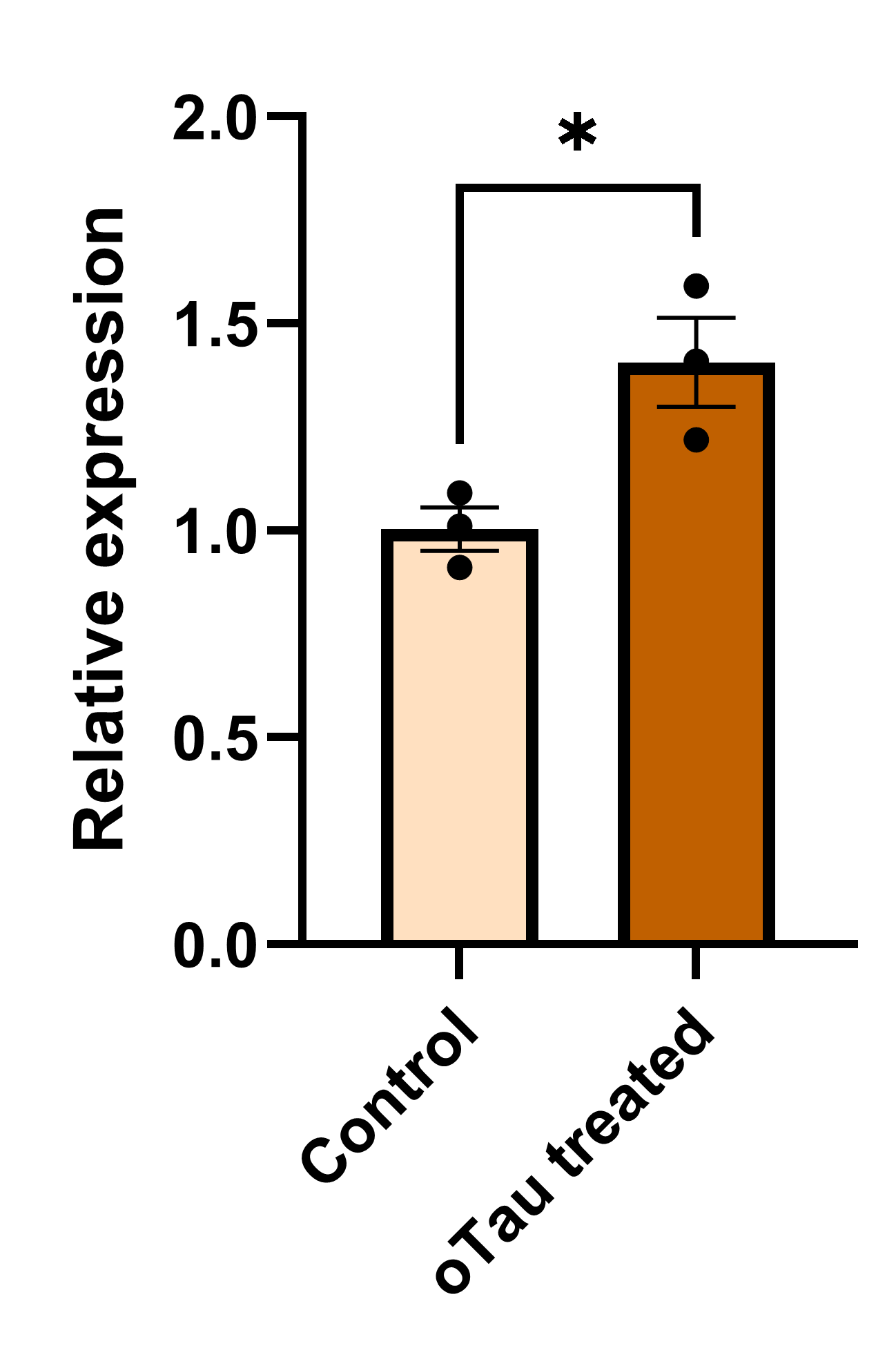


**Supplement figure 14.** (A) Immunoblot analysis of mTOR in 3D assembloids model of tauopathy under different conditions. (n=3). (B) Relative circPDE4B expression measured by qPCR in 3D assembloids comparing control (vehicle-treated) and oTau-treated conditions (150 assembloids pooled per condition; n = 3 biological replicates). Data are presented as mean ± SEM. Statistical significance was determined by one-way ANOVA for (A) and unpaired Student’s t-test for (B). p < 0.05 (*), **p < 0.001.


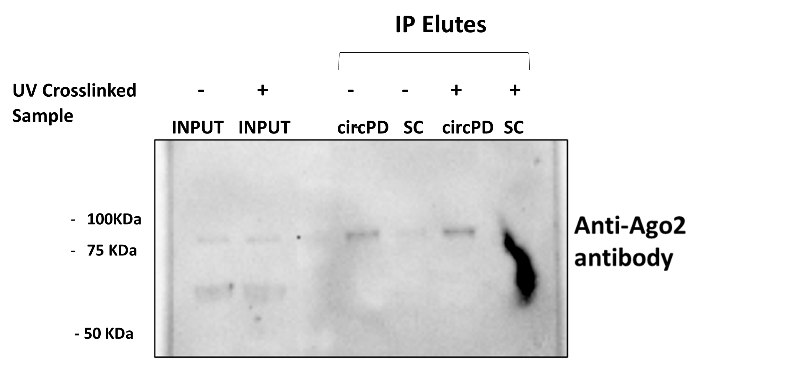

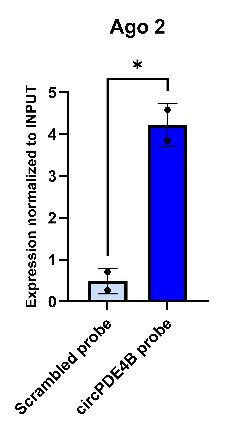


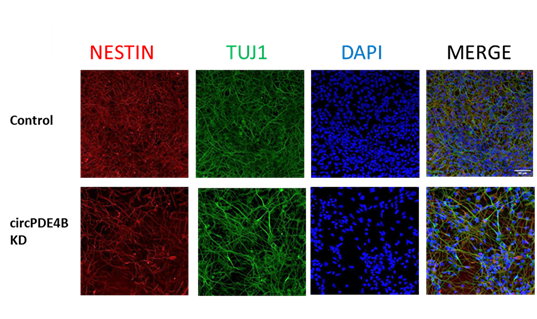
**Supplement figure 15.** Immunoblot showing AGO2 enrichment in circPDE4B probe immunoprecipitation compared with scrambled probe control. Data are presented as mean ± SE. P-values are indicated; *p < 0.05, by unpaired student-t test.

**A**


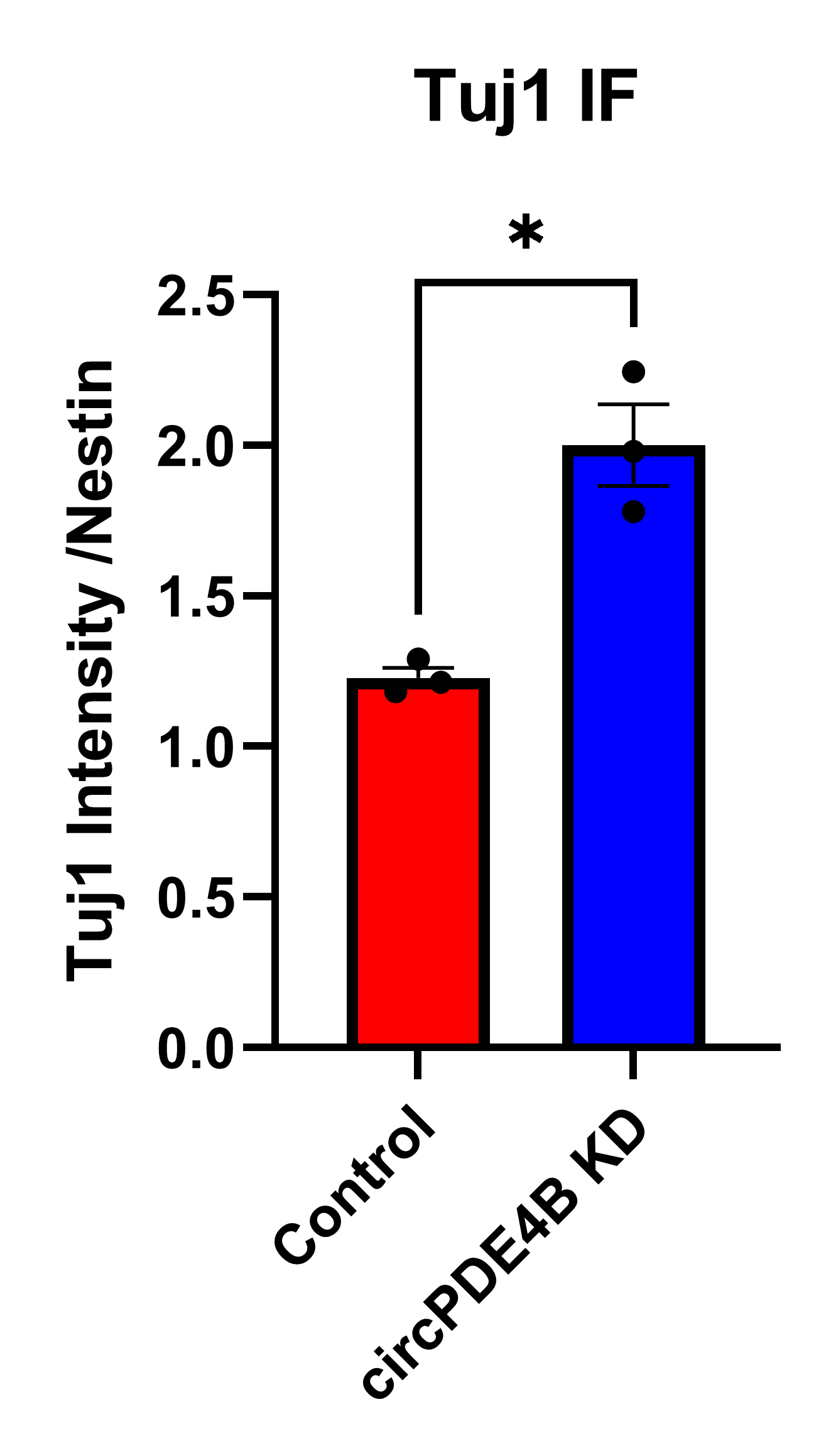


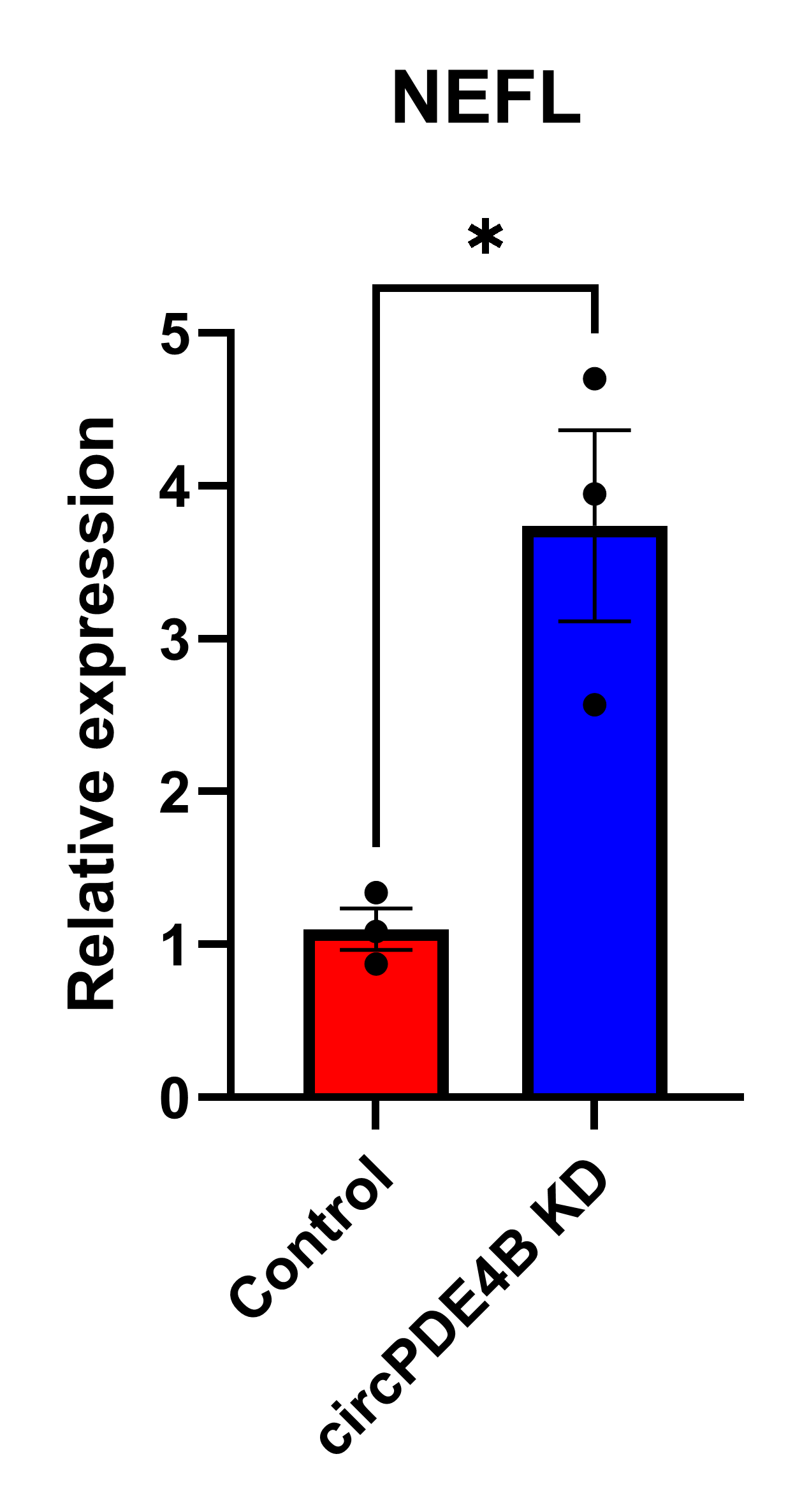

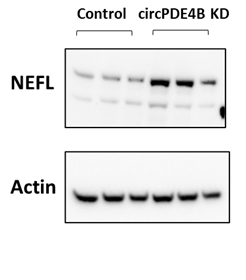
**B**

**Supplement Figure 16:** (A) Immunofluorescence staining for Tuj1 reveals higher expression in circPDE4B knockdown cells (red: Nestin; green: Tuj1; blue: DAPI). (B) Immunoblot analysis shows increased NEFL expression in circPDE4B knockdown cells. N = 3 biological replicates. Data are presented as mean ± SEM. P-values are indicated; *p < 0.05 by unpaired Student's t-test.

**
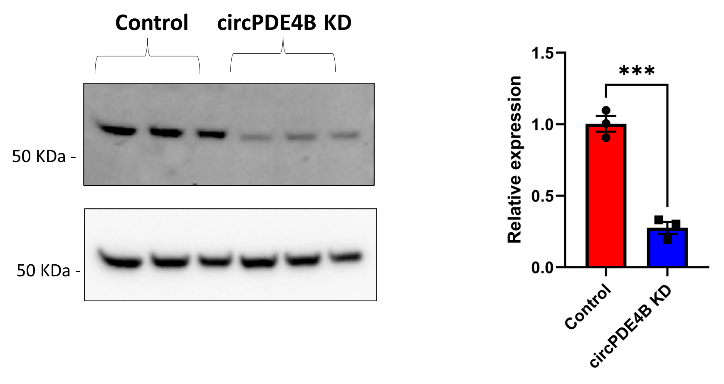
**

**Supplement Figure 17.** Immunoblot showing Myc decrease in circPDE4B downregulated cells (n=3). Data are presented as mean ± SE. P-values are indicated; ***p < 0.005 by unpaired student-t test.
